# Evolution of Anabaenopeptin Peptide Structural Variability in the Cyanobacterium *Planktothrix*

**DOI:** 10.3389/fmicb.2017.00219

**Published:** 2017-02-16

**Authors:** Elisabeth Entfellner, Mark Frei, Guntram Christiansen, Li Deng, Jochen Blom, Rainer Kurmayer

**Affiliations:** ^1^Research Institute for Limnology, University of InnsbruckMondsee, Austria; ^2^Miti Biosystems GmbH, Max F Perutz LaboratoriesWien, Austria; ^3^Institute of Virology, Helmholtz Zentrum MünchenMünchen, Germany; ^4^Bioinformatics and Systems Biology, Justus-Liebig-UniversityGiessen, Germany

**Keywords:** cyanotoxins, cyanoHABs, natural products, chemotype, ecotype, speciation, horizontal gene transfer, gene deletion

## Abstract

Cyanobacteria are frequently involved in the formation of harmful algal blooms wherein, apart from the toxic microcystins, other groups of bioactive peptides are abundant as well, such as anabaenopeptins (APs). The APs are synthesized nonribosomally as cyclic hexapeptides with various amino acids at the exocyclic position. We investigated the presence and recombination of the AP synthesis gene cluster (*apnA-E*) through comparing 125 strains of the bloom-forming cyanobacterium *Planktothrix* spp., which were isolated from numerous shallow and deep water habitats in the temperate and tropical climatic zone. Ten ecologically divergent strains were purified and genome sequenced to compare their entire *apnA-E* gene cluster. In order to quantify *apn* gene distribution patterns, all the strains were investigated by PCR amplification of 2 kbp portions of the entire *apn* gene cluster without interruption. Within the 11 strains assigned to *P. pseudagardhii, P. mougeotii*, or *P. tepida* (Lineage 3), neither *apnA-E* genes nor remnants were observed. Within the *P. agardhii*/*P. rubescens* strains from shallow waters (Lineage 1, 52 strains), strains both carrying and lacking *apn* genes occurred, while among the strains lacking the *apnA-E* genes, the presence of the 5′end flanking region indicated a gene cluster deletion. Among the strains of the more derived deep water ecotype (Lineage 2, 62 strains), *apnA-E* genes were always present. A high similarity of *apn* genes of the genus *Planktothrix* when compared with strains of the genus *Microcystis* suggested its horizontal gene transfer during the speciation of *P. agardhii*/*P. rubescens*. Genetic analysis of the first (A_1_-) domain of the *apnA* gene, encoding synthesis of the exocyclic position of the AP molecule, revealed four genotype groups that corresponded with substrate activation. Groups of genotypes were either related to Arginine only, the coproduction of Arginine and Tyrosine or Arginine and Lysine, or even the coproduction of Arginine, Tyrosine, and Lysine in the exocyclic position of the AP-molecule. The increased structural diversity resulted from the evolution of *apnA* A_1_ genotypes through a small number of positively selected point mutations that occurred repeatedly and independently from phylogenetic association.

## Introduction

**Figure 1:**
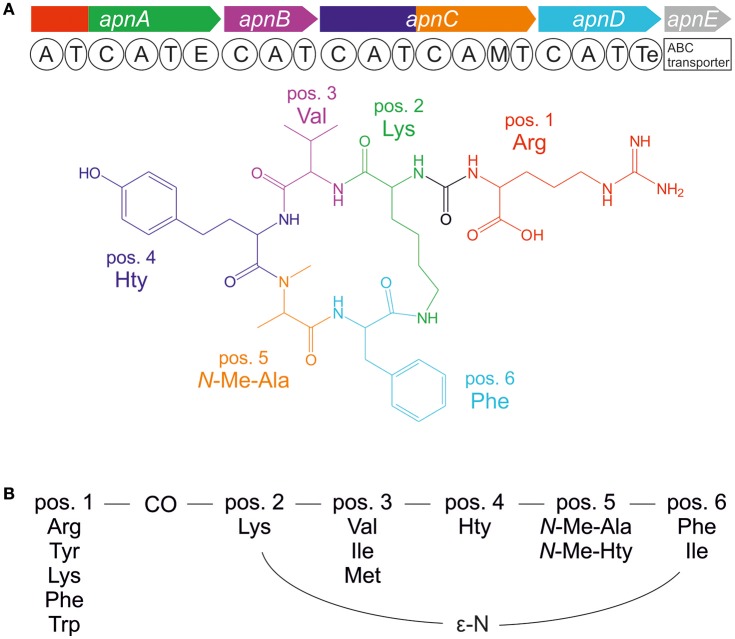
**(A)** Anabaenopeptin synthesis gene cluster and resulting molecular structure of anabaenopeptin B ([M+H]^+^ 837) and **(B)** amino acid variation of anabaenopeptins as observed in the genus *Planktothrix*.

The bloom-forming cyanobacteria of *Planktothrix agardhii* and *P. rubescens* are frequently involved in cyanotoxin production in lakes and reservoirs. Besides the toxic heptapeptide microcystin, a number of additional bioactive oligopeptides have been elucidated from *Planktothrix* spp., (e.g., Kurmayer et al., 2016). In particular, the anabaenopeptins (APs) show an impressive diversity in bioactivity. For example, while some AP structural variants inhibit protein phosphatase 1 and 2A, others have serine proteases inhibition activity such as chymotrypsin and trypsin, or they are potent inhibitors of carboxypeptidase A (e.g., in Spoof et al., 2016) and other metallocarboxypeptidases (Halland et al., 2015). APs are cyclic hexapeptides consisting of five amino acid residues forming a ring (pos. 2–6) and an exocyclic residue (pos. 1), which is connected to the ring through an ureido bond (Figure 1). While the D-Lys in pos. 2 and the ureido bond of the AP structure are conserved motifs, different amino acids are found in all other positions of the AP molecule resulting in numerous structural variants (e.g., in Spoof et al., 2016). The first AP structural variants A and B were described from *Anabaena flos-aquae* (Harada et al., 1995). Other cyanobacteria genera known as prominent AP producers include the planktonic genera *Microcystis* (e.g., Williams et al., 1996; Fastner et al., 2001), or *Nodularia* (e.g., Fujii et al., 1997) but also benthic genera such as *Lyngbya* (e.g., Zi et al., 2012) and *Schizothrix* (e.g., Reshef and Carmeli, 2002). In general, the AP peptides are the most abundant besides the microcystins in waterbodies of the temperate climate region (Halstvedt et al., 2008; Gkelis et al., 2015). Typically, cellular contents up to 0.5% dry weight are reported in isolated strains (0.9–10 μg AP mg^−1^ dry weight), (Kosol et al., 2009), and in field samples high concentrations >1 mg L^−1^ have been observed (e.g., Gkelis et al., 2015).

APs are produced by nonribosomal peptide synthetases (NRPS) that follow a stepwise synthesis pathway using (non) proteinogenic amino acids as substrate (Christiansen et al., 2011). Following the thio-template mechanism, the amino acid substrates are activated as aminoacyl adenylate and condensed to the growing peptide chain. Minimum modules consist of adenylation (A), thiolation (T), and condensation (C) domains. Additional enzyme domains catalyze the epimerization of the conserved D-Lys in pos. 2 and *N*-methylation in pos. 5 (Figure 1; Christiansen et al., 2011). The first A-domain of ApnA (ApnA A_1_) activates different amino acids occurring at the variable exocyclic pos. 1. Molecular biological and evolutionary analysis revealed that single point mutations within the first A-domain can result in a remarkable substrate promiscuity since the two chemically divergent amino acids arginine and tyrosine were activated with high selectivity and comparable efficiency (Christiansen et al., 2011). Based on these results, those particular enzyme domains were crystallized and the structural basis of substrate activation describing a bispecific A-domain was elucidated (Kaljunen et al., 2015).

Both of these previous studies, however, were based on the investigation of only a small number of selected strains. In the present study, we aimed to qualitatively and quantitatively analyze the recombination phenomena leading to *apnA-E* gene cluster evolution in the genus *Planktothrix*. *Planktothrix* occurs in shallow and deep water ecosystems of the temperate and tropical climatic zones. Recent phylogenetic and ecological analysis has defined a number of lineages representing ecological diversification (Gaget et al., 2015; Kurmayer et al., 2015). In a first attempt, we compared the *apn* gene cluster sequence and its flanking regions from 10 ecologically divergent strains for which the genomes were sequenced. In addition, we examined all other *Planktothrix* strains for the *apn* gene cluster presence/absence and recombination. In a second step, we analyzed the nucleotide variation of the *apnA* A_1_-domain and the resulting AP peptide structural variation to identify the functional consequences of genetic structural recombination in 89 AP-producing strains. If a relationship between *apnA* A_1_-genotypes and the occurrence of AP variants exists, the ecological dynamics of specific *apnA* A_1_ genotypes can be followed to investigate the evolution of AP synthesis in our water bodies.

## Materials and methods

### Organisms

In total, 125 clonal *Planktothrix* spp. strains, isolated from deep and shallow freshwater habitats, were analyzed in this study (Supplementary Table S1). One hundred twelve strains were previously characterized and assigned to phylogenetic lineages by multilocus sequence analysis (MLSA) and 13 additional strains were added into this earlier phylogeny (Kurmayer et al., 2015). The strains were grown under sterile conditions in BG11 medium with low light intensity (5–10 μmol m^−2^ s^−1^ Osram Type L30W/77 Fluora, 16/8 h light-dark cycle, 15° or 23°C).

### DNA isolation

Cells from cultures were harvested by centrifugation and washed in TE buffer (10 mM Tris, 1 mM EDTA, pH 8.0 with HCl). DNA was extracted according to Reinard (2008). In brief, the cell pellet was suspended in 0.5 ml of CTAB solution (0.1 M cetyltrimethylammonium bromide, 1.4 M NaCl, 0.1 M Tris, 0.02 M EDTA), incubated at 65°C (30 min) followed by enzymatic treatment (proteinase K, 1 mg ml^−1^, 60°C, 60 min and RNase A, 0.2 mg ml^−1^, 20°C) and DNA extraction with chloroform/isoamyl alcohol (24:1, v/v). The DNA was precipitated by mixing the aqueous phase with two volume parts of CTAB precipitation solution (15 mM CTAB, 40 mM NaCl, pH 7.0) at 4°C (1 h) to purify the DNA from polysaccharides. The DNA pellet was dissolved in 1.2 M NaCl solution and extracted again with chloroform/isoamyl alcohol (24:1, v/v). Finally, the DNA was precipitated with 0.6 volume parts isopropanol. The pellet was washed with 70% (v/v) ethanol and the DNA was dissolved in 50 μl EB buffer (Qiagen, Hilden, Germany).

### Genome sequencing

Ten *Planktothrix* strains from different phylogenetic lineages were axenized (Rippka, 1988). Purity was tested and confirmed using DAPI (4,6-diamidino-2-phenylindole) staining for contaminant bacteria on membrane filters (0.2 μm pore size) and epifluorescence microscopy. High molecular weight DNA was extracted from cells by grinding in liquid nitrogen, purified using anion-exchange columns (Qiagen) according to the manufacturer's protocol and used for single molecule real-time (SMRT) sequencing (GATC Biotech, Constance, Germany). The sequencing efforts aimed to obtain a hundredfold coverage resulting in a relatively low number of contigs (Table 1). The genome sequences were annotated automatically using GenDB (Meyer et al., 2003) with the reference genome of *P. agardhii* NIVA-CYA 126/8 (Christiansen et al., 2014), access. no. CM002803.1. For all strains with sequenced genome, the automatically annotated chromosomal region carrying the *apnA-E* gene cluster and/or its flanking regions (approx. 300 kbp) have been manually curated and submitted to DDBJ/EMBL/GenBank under the accession no. KU665235-KU665242.

**Table 1:** **Genome characteristics of ***Planktothrix*** spp. Strains**.

**Taxonomic affiliation**	**Phylogenetic lineage**	**Strain name**	**Mean coverage**	**Number of contigs**	**Putative plasmids (kbp)**	**Chrom. size (mbp)**	**Length of *apnA-E* gene cluster (bp)**	**% Identity of *apnA-E* genes to NIVA-CYA 98^c^**
*P. agardhii*^a^	1	NIVA-CYA 126/8	93	7	120, 50	4.8	23,949	86.4
*P. agardhii*^a^	1	PCC 7805	113	4	153	4.8	nd	na
*P. agardhii*^a^	1	No. 66	88	19	94, 44	4.9	23,933	96.1
*P. agardhii*^a^	1A	No. 365	77	23	80, 56, 40, 23	4.7	23,957	96.3
*P. rubescens*^a^	2	No. 82	78	16	82, 79, 68, 14	5.5	23,930	98.8
*P. rubescens*^a^	2	PCC 7821	78	5	93, 79, 48, 13	5.5	23,930	100
*P. rubescens*^a^	2	No. 108	91	23	nd	5.6	23,930	98.5
*P. agardhii*^a^	2A	No. 758	86	6	nd	5.6	23,930	98.0
*P. tepida*^b^	3	PCC 9214	60	7	84, 50	6.6	nd	na
*P. mougeotii*^a^	3	CCAP 1460/6	66	13	nd	6.6	nd	na

a according to Suda et al. (2002);

b according to Gaget et al. (2015);

c *access no. AM990463.2 (Rounge et al., 2009)*.

*nd, not detected; na, not applicable*.

### PCR analysis and sequencing

For the MLSA of newly sequenced strains (*n* = 13), seven gene loci and intergenic spacer (IGS) regions, including the 16S rDNA (301 bp), 16S rDNA-internal transcribed spacer (ITS) region (312 bp), phycocyanin (PC)-IGS (204 bp), *psa*A, and *psa*B photosynthesis protein (PSA)-IGS (548 bp), RNaseP gene (299 bp), large subunit of the ribulose bisphosphate carboxylase/oxygenase and *rbc*X (*rbc*LX)-IGS (336 bp), and *rpo*C (492 bp) were amplified by PCR and sequenced (Kurmayer et al., 2015), GenBank accession no. KU574075–KU574122.

In order to estimate the presence/absence of the *apnA-E* gene cluster in all 125 strains, 17 primer pairs were designed from the reference gene clusters of NIVA-CYA 126/8 (Christiansen et al., 2011; access. no. EF67686) and NIVA-CYA 98 (Rounge et al., 2009; access. no. AM990463.2) and used to amplify overlapping fragments without interruption (approx. 2 kbp for each amplicon), (Supplementary Table S2). Each PCR reaction mixture had a total volume of 10 μl, containing 2 μl (5 ×) Phusion HF Buffer (Thermo Scientific, Vienna, Austria), 500 nM of each primer, 200 μM of each deoxynucleotide triphosphate (Thermo Scientific), 0.1 U of Phusion High-Fidelity DNA Polymerase (Thermo Scientific), and 10 ng of DNA. PCR amplification was performed under the following conditions: Initial denaturation at 98°C for 1 min; followed by 35 cycles of denaturation at 98°C for 10 s, annealing at a variable temperature for 15 s and elongation at 72°C. The PCR amplicon size was determined using gel electrophoresis (0.8% agarose gels in 0.5 × Tris-borate-EDTA buffer and visualized using Midori Green). For the sequencing of adenylation domains, PCR products were extracted from the agarose gel using a gel extraction kit (QIAquick, Gel Extraction Kit, Qiagen) and sequenced (Eurofins Genomics, Ebersberg, Germany). Nucleotide sequences were submitted to GenBank under accession no. KU639970–KU640047.

### Nucleotide sequence analysis

For the MLSA analysis of all 125 strains, the sequences of the seven gene loci were concatenated. A maximum likelihood tree was calculated using default adjustments in Mega 6.0 (phylogeny test: bootstrap method, 1,000 replicates; substitution model: Tamura 3-parameter model; gaps included). We used the BLASTn algorithm to identify *apn* genes or homologs in other cyanobacteria genera (access. no. AQPY01000292.1; AZYY01000200.1; CAIH01000020.1; CAIQ01000336.1; CP001037.1; CP003284.1; CP007203.2; JHEG02000042.1; KV757545.1; LJOQ01000025.1; LJOP01000059.1; LWAJ01000278.1) and compared the individual genes by calculating the evolutionary divergence as well by phylogenetic analysis using maximum likelihood (Mega 6.0). The *apnA* A_1_ sequences from all 89 AP-producing strains (core motive A4-A6, 510 bp) were aligned and maximum likelihood (ML) was used to construct a phylogenetic tree (Mega 6.0). Default parameters, such as a constant rate variation among the sites as well as a fixed transition/transversion ratio, have been used. In general, sites were not weighted. Statistical significance of the branches was estimated by bootstrap analysis generating 1,000 replicates of the original data set. ApnA A_1_ domain selectivity was predicted using established bioinformatics tools based on the specificity-conferring code defined by Stachelhaus et al. (1999) and the residues within 8 Å around the substrate as defined by Rausch et al. (2005).

The ratio of non-synonymous (dN) and synonymous (dS) *apnA* A_1_ substitution rates per codon site was determined using maximum likelihood estimates as implemented in the PAML package (Version 3.15; Yang, 1997). NS-sites models were applied to identify the sites under potential positive selection. A likelihood ratio test (LRT, *df* = 2) was constructed to compare the likelihood of the phylogenetic tree, calculated under two different type of models: (1) the null models M1 (nearly neutral), M7 (beta), which do not allow for positively selected sites (dN/dS ≤ 1), and (2) the alternative models M2 (positive selection), M8 (beta & dN/dS > 1), which adds an additional site class that accounts for positive selection (dN/dS > 1). When the LRT was found to be significant, the Bayes empirical Bayes (BEB) approach was used to calculate posterior probabilities that a site comes from the site class with dN/dS > 1 (Yang et al., 2005).

### Anabaenopeptin peptide structural identification

Cells were harvested on glass fiber filters (BMC, Ederol, Vienna, Austria) and dried biomass was extracted in 50% (v/v) aqueous methanol on ice according to Kosol et al. (2009). AP structural variants were separated by HPLC (HP 1100, Agilent) using a linear water/acetonitrile (0.05% trifluoroacetic acid) gradient from 80:20 to 50:50 in 45 min at a flow rate of 1 ml min^−1^ and 30°C oven temperature, LiChrospher 100 octyldecyl silane (ODS) (5 μm particle size) and LiChroCART 250-4 cartridge system (Merck, Darmstadt, Germany) as described previously (Kosol et al., 2009). The HPLC system was coupled to an Electrospray Ionisation (ESI) Mass Spectrometer ion trap (amaZonSL, Bruker) operating in positive ion mode. Nitrogen was used as sheath gas (43 psi, 8 L/min, 300°C) and helium was used as auxiliary gas. Capillary voltage was set to 5 kV. Under these conditions, the following AP structural variants were detected: AP B (836.5 Da), AP A (843.4 Da), AP F (850.5 Da), Oscillamide (Osc) Y (857.4 Da), AP C (808.4 Da), AP D (827.4 Da), AP 908 (908.5 Da), AP 915 (915.5 Da), Osc B (868.4 Da), and ferintoic acid A (866.4 Da), (Harada et al., 1995; Sano and Kaya, 1995; Williams et al., 1996; Shin et al., 1997; Sano et al., 2001; Fujii et al., 2002; Okumura et al., 2009; Supplementary Figures S1, S2). Unknown AP structural variants were assigned according to specific fragmentation patterns (Supplementary Tables S3, S4). In general, for each strain, 2–7 mg of dry weight were extracted. The limit of detection for AP B was 10 ng (corresponding to 0.005–0.0014% of dry weight). All AP structural variants that contributed ≥5% of peak area compared with the most abundant AP structural variant (extracted ion chromatogram) were recorded.

## Results

### Distribution of the anabaenopeptin synthesis genes among the genus *planktothrix*

**Figure 2:**
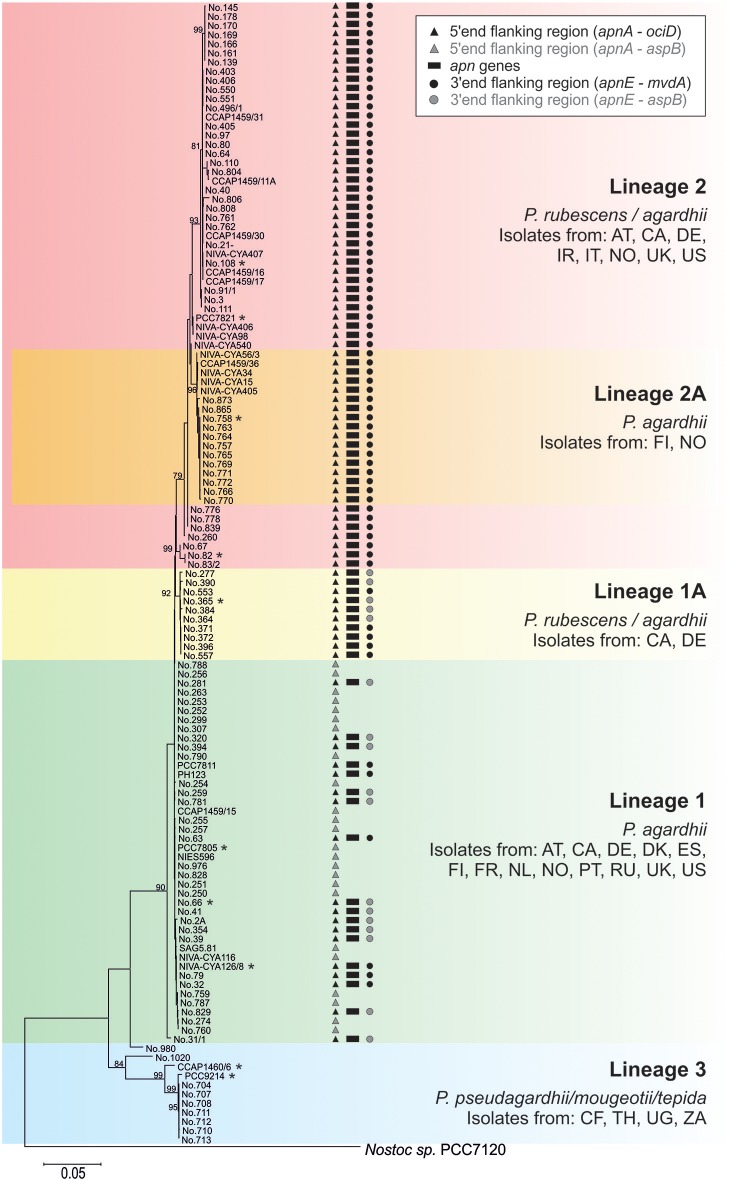
**Phylogenetic affiliation of the 125 ***Planktothrix*** strains analyzed for the ***apnA-E*** gene cluster (genome sequenced strains are marked with a star)**. The Maximum likelihood tree was constructed from seven gene loci or intergenic spacer regions. Numbers at nodes indicate the percent bootstrap frequency (1,000 replicates). Only bootstrap values >70% are shown. The tree was rooted using *Nostoc* sp. strain PCC 7120. Countries of origin are indicated by the two-letter code.

**Figure 3:**
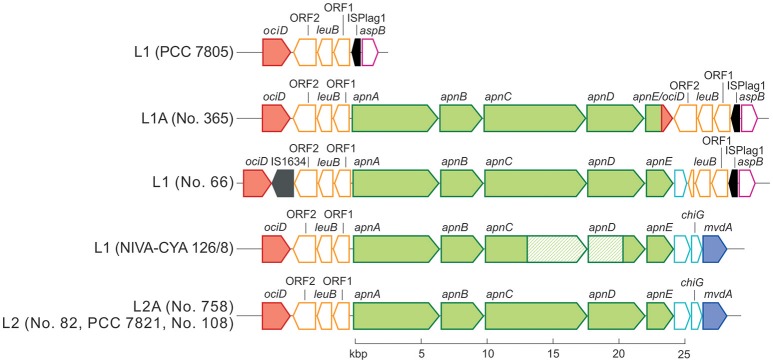
**Schematic view of the ***apnA-E*** gene cluster (green color) and flanking regions**. The microviridin synthesis gene cluster (*mvdA-F*) and the cyanopeptolin synthetase genes (*ociA-D*) are shown in blue and red, each. Identical sequences are indicated by the same color. Transposable elements are marked in black.

Phylogenetic analysis of all 125 *Planktothrix* strains revealed a diversification comprising three major lineages: Lineage 1 and Lineage 2 represented only the strains assigned to *P. agardhii*/*P. rubescens* and Lineage 3 represented *P. pseudagardhii, P. mougeotii*, and *P. tepida* (Figure 2). While strains of Lineage 1 were mainly isolated from shallow lakes, most strains of Lineage 2 were isolated from deep lakes in the temperate climatic zone. Strains of Lineage 3 originated from lakes in the tropical climatic zone. From these ecologically divergent lineages, 10 strains were purified and their genomes sequenced (Table 1). Following assembly and annotation, the *apnA-E* gene cluster structure and flanking regions were elucidated. As reported previously, the *apnA-E* gene cluster spans 24 kbp and encodes six NRPS modules (two modules in *apnA*, one in *apnB*, two in *apnC*, and one in *apnD*) as well as an ATP binding cassette (ABC) transporter (*apnE*), (Christiansen et al., 2011). Except for strain PCC 7805, all sequenced genomes of *P. agardhii*/*P. rubescens* strains were found to contain the *apnA-E* gene cluster in the chromosome (Figure 3). Notably, in all sequenced genomes of strains of Lineage 1+2 (including the PCC 7805 genome), the 5′end flanking region of the *apnA-E* gene cluster was found to be similar (alignment 6,682 bp, evolutionary divergence 0.010–0.035), and showed a linkage to the cyanopeptolin synthesis gene cluster as described by Rounge et al. (2009). Among the Lineage 1+2 strains with genome sequences, the 3′end flanking region was found variable and either contained another gene cluster encoding microviridin synthesis (Philmus et al., 2008)—thereby forming a meta peptide synthesis gene cluster—or the sequence reported for strain PCC 7805 (strains no. 66 and no. 365). In contrast, strains of Lineage 3 (*P. pseudagardhii, P. mougeotii, P. tepida*) did not contain any sequence related to the *apnA-E* gene or flanking region. For all other 115 strains, the presence of the entire *apnA-E* gene cluster was tested using the PCR amplification of ~2 kbp fragments without interruption (Supplementary Table S2, Figure S3). In total, 90 out of 125 strains (72%) carried the AP synthesis operon. Among Lineages 2 and 2A, all strains were found to contain the *apn* gene cluster (Figure 2), with only strain CCAP 1459/31 showing a 381 bp-deletion within the A-domain of *apnB* resulting in the inactivation of AP synthesis. The *apnA-E* genes also occurred among the strains of Lineage 1 (43% of strains) and 1A (all strains). Corresponding with the genomic results, no evidence for *apnA-E* gene presence was found among the strains of Lineage 3. The PCR assay amplifying the 5′end flanking region (*ociD-apnA*) gave the expected specific PCR product (4,719 bp in reference strain NIVA-CYA 126/8) for all of the 90 *apnA-E* positive strains of Lineage 1 and 2. Three strains (no. 66, 63, 41) showed a longer PCR product due to the insertion of a sequence similar to IS1634, 2,063 bp (access. no. JX 134573). Interestingly, strains lacking the *apnA-E* gene cluster from Lineage 1 (*n* = 24) gave the expected PCR product of the 5′end flanking region (5,073 bp, *ociD-aspB*). In contrast, for the strains of Lineage 3, no PCR products were obtained. The DNA fragment of the 3′end flanking region was amplified less consistently among *apnA-E* positive strains (77 out of 90). All the strains from Lineage 2 and 2A showed a specific PCR product with the expected size (4,411 bp in reference strain NIVA-CYA 126/8) suggesting the phylogenetic fixation of the meta peptide synthesis gene cluster. However, 18 strains from Lineage 1 (1A) failed to show this PCR product, implying that according to genomic information of strains no. 66 and 365, the *mvdA-F* gene cluster was not associated with the *ociA-D* and *apnA-E* gene cluster (Supplementary Table S1). In summary, the *apnA-E* gene cluster occurred among all the strains of Lineage 2 (2A) and showed almost identical flanking regions. Among Lineage 1 (1A) strains both carrying and lacking the *apn* gene cluster were found, but all of them contained at least the 5′end flanking region, suggesting a potential deletion.

### Comparison of anabaenopeptin synthesis gene cluster genes and flanking regions among cyanobacteria

**Figure 4:**
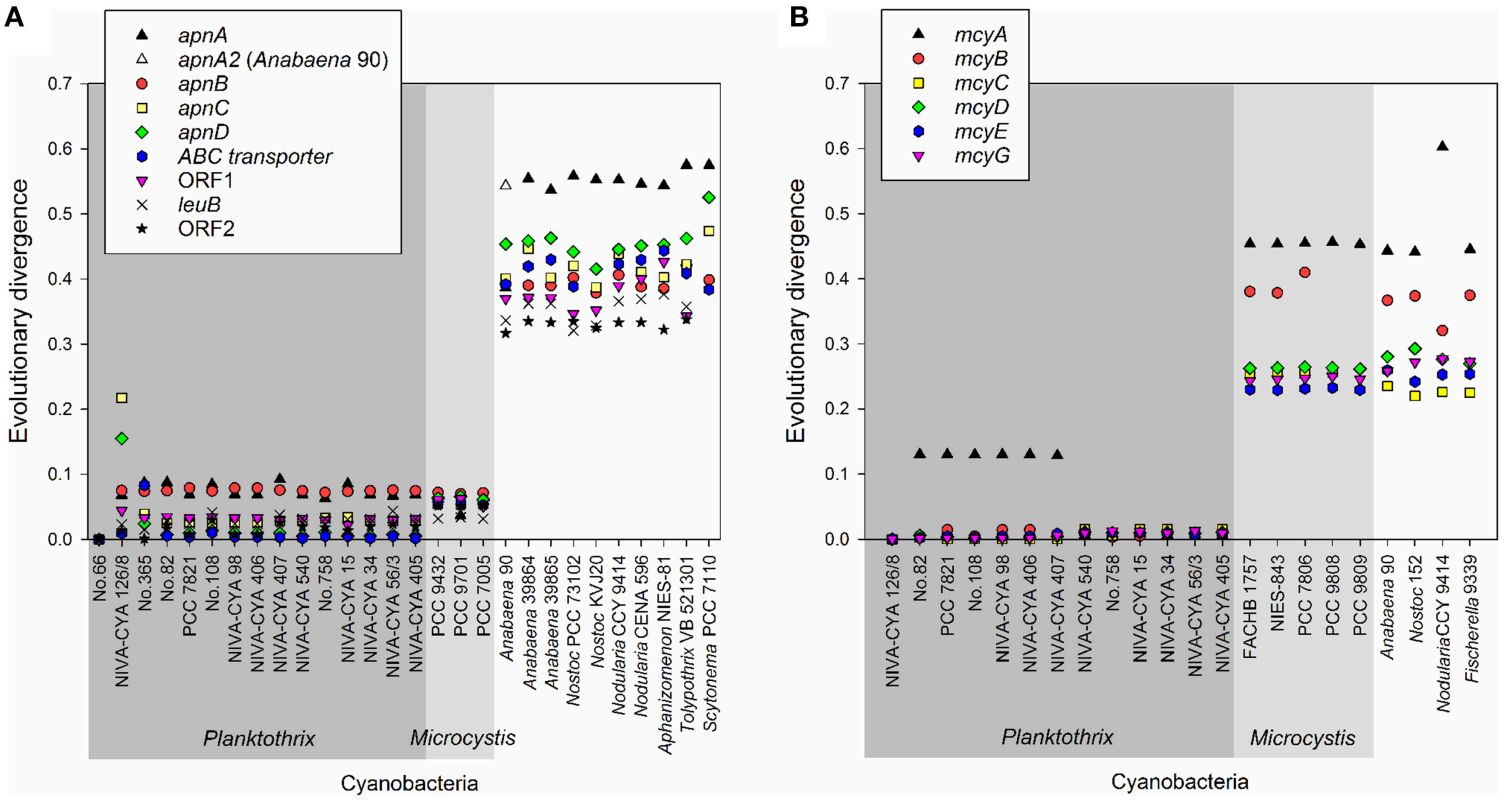
**(A)** Evolutionary divergence (nucleotide variability) between the individual *apnA-E* genes of *Planktothrix* strain no. 66 when compared with other strains *Planktothrix* and various cyanobacteria; **(B)** Evolutionary divergence (nucleotide variability) of individual *mcyA-G* genes as calculated from various cyanobacteria. *Planktothrix* strain NIVA-CYA 126/8 was used as a reference.

Using the BLASTn algorithm against the Genbank nucleotide database, *apn* genes or homologs were found for eight genera: *Planktothrix, Microcystis*, and the heterocystous genera (*Anabaena, Nostoc, Nodularia, Aphanizomenon, Tolypothrix, Scytonema*), (Figure 4A). With the exception of the large recombination within *apnC/D* observed for the strain NIVA-CYA 126/8, the *apnA-E* genes showed low evolutionary divergence (≤0.1) within the genus *Planktothrix*. Notably, the nucleotide divergence remained low compared to the *apn* genes of *Microcystis*, but it increased to >0.3 when comparing with *apn* genes of other genera. Phylogenetic trees grouped *apnA-E* genes from both genera *Planktothrix* and *Microcystis* into one branch while the *apnA-E* genes of other heterocystous genera formed a separate lineage (Supplementary Figures S4–S8). Notably, for *apnA* and *apnB* genes, *Planktothrix* strain no. 66 was placed within *Microcystis*. The high similarity of *apnA-E* genes between *Planktothrix* and *Microcystis* is in contrast to other NRPS genes, such as the microcystin synthesis gene cluster, which is phylogenetically differentiated between *Planktothrix* and *Microcystis* (Figure 4B). We conclude that the high similarity of *apn* genes between *Planktothrix* and *Microcystis* stems from horizontal gene transfer.

### Recombination of anabaenopeptin synthesis genes among *planktothrix*

Within the strains with sequenced genome, the *apnA-E* gene clusters were 96–100% similar to *apnA-E* genes from the strain NIVA-CYA 98 (Rounge et al., 2009). Only the strain NIVA-CYA 126/8 showed a large recombination of parts of the second module of *apnC* (3,235 bp) comprising the condensation domain *apnC* C_2_ and the A-domains *apnC* A_2_ and *apnD* A (795 bp), which has been previously described (Christiansen et al., 2011). Using PCR amplification, only two other strains of Lineage 1 (nos. 259, 281) showed this large recombination, resulting in the same amino acid variation in the AP molecule as reported for the strain NIVA-CYA 126/8 (Supplementary Table S1; Christiansen et al., 2011).

Notably, the genome of strain no. 365 showed a fusion of the two ABC transporter genes *apnE* (1,581 bp) with *ociD* (483 bp), (Figure 3) which is part of the cyanopeptolin synthesis gene cluster (Rounge et al., 2007), at the conserved Walker B motif (amino acid position 526–533 in the *apnE* of the strain NIVA-CYA 126/8; Pearson et al., 2004). This recombination was found in five additional strains from Lineage 1 and 1A (no. 354, 364, 384, 390, 394; Supplementary Table S1). No functional consequence on AP production was observed due to this recombination. In summary, the recombinations of larger fragments affecting A-domains *apnC* A_2_/*apnD* A occurred only rarely.

### Genetic diversity of the A-Domain *apnA* A_1_ correlates to structural variation in the anabaenopeptin molecule

**Figure 5:**
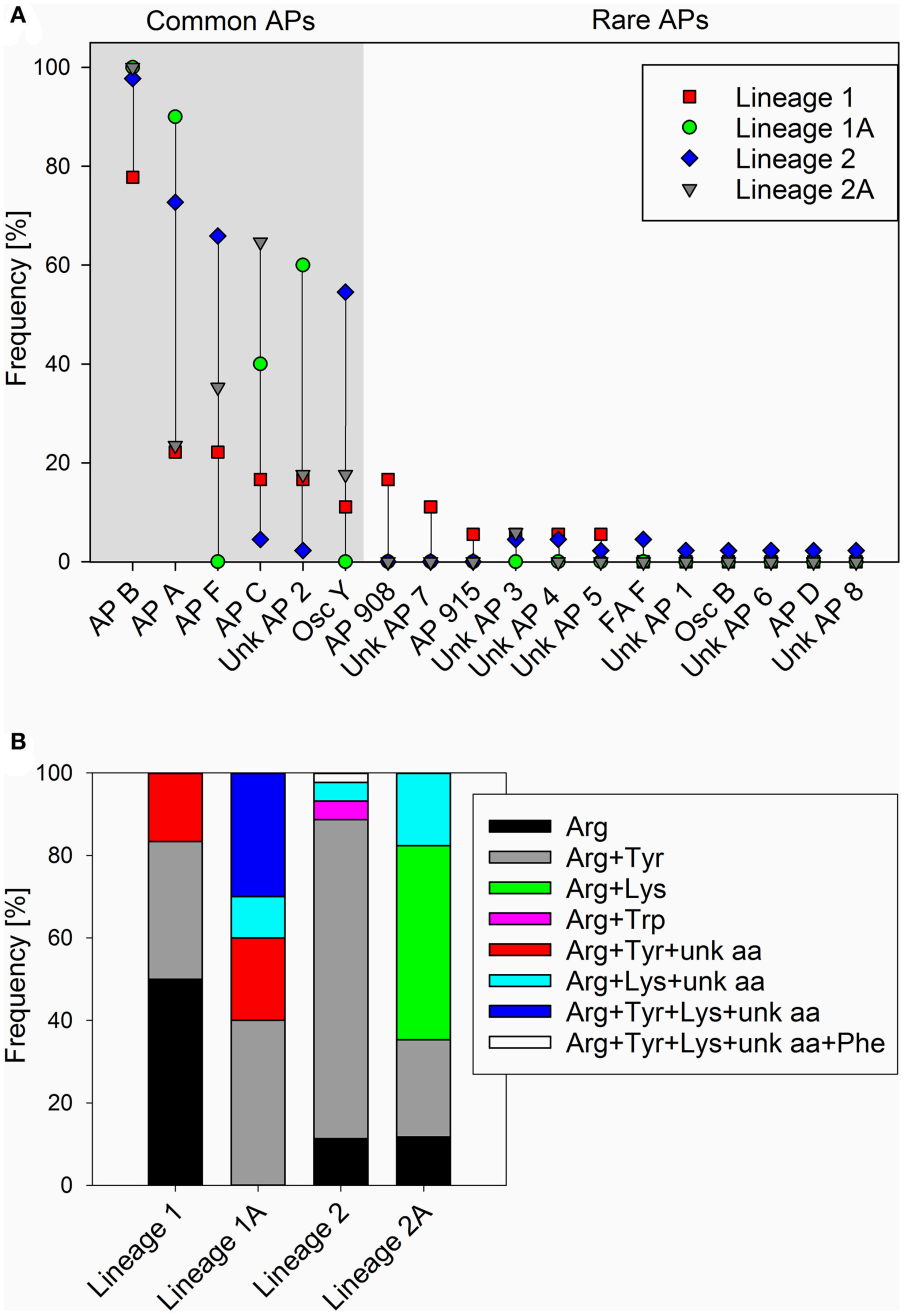
**(A)** Frequency (proportion) of anabaenopeptin structural variants among *Planktothrix* phylogenetic lineages 1 (1A), 2 (2A); **(B)** Frequency (proportion) of co-production of various amino acids in exocyclic pos. 1 of AP structural variants.

For all 89 strains carrying the full *apnA-E* gene cluster, we observed the synthesis of at least one AP structural variant (Supplementary Table S1). The AP structural variant with Arg (AP B, [M+H]^+^ 837) in exocyclic pos. 1 of the AP molecule occurred most frequently (93%). Other structural variants with Arg (AP F, [M+H]^+^ 851) or Tyr (AP A, [M+H]^+^ 844), (Osc Y, [M+H]^+^ 858), or Lys (AP C, [M+H]^+^ 809) in exocyclic pos. 1 of the AP molecule occurred with a median proportion of 45, 55, 33, and 22%, respectively (Figure 5A). In addition, other AP structural variants occurred in a few strains only, e.g., AP D with Phe in pos. 1 ([M+H]^+^ 828) or ferintoic acid A with Trp in pos. 1 ([M+H]^+^ 867). Unknown APs were identified by comparing fragmentation patterns with those of known AP structural variants. With regard to the co-production of AP structural variants differing in amino acid (aa) composition in pos. 1 the following chemotypes were observed: Arg (*n* = 16), Arg + Tyr (*n* = 48), Arg + Lys (*n* = 8), Arg + Trp (*n* = 2), Arg + Tyr + unknown aa (*n* = 3), Arg + Lys + unknown aa (*n* = 9), Arg + Tyr + Lys + unknown aa (*n* = 2), Arg + Tyr + Lys + unknown aa + Phe (*n* = 1), (Figure 5B).

**Figure 6:**
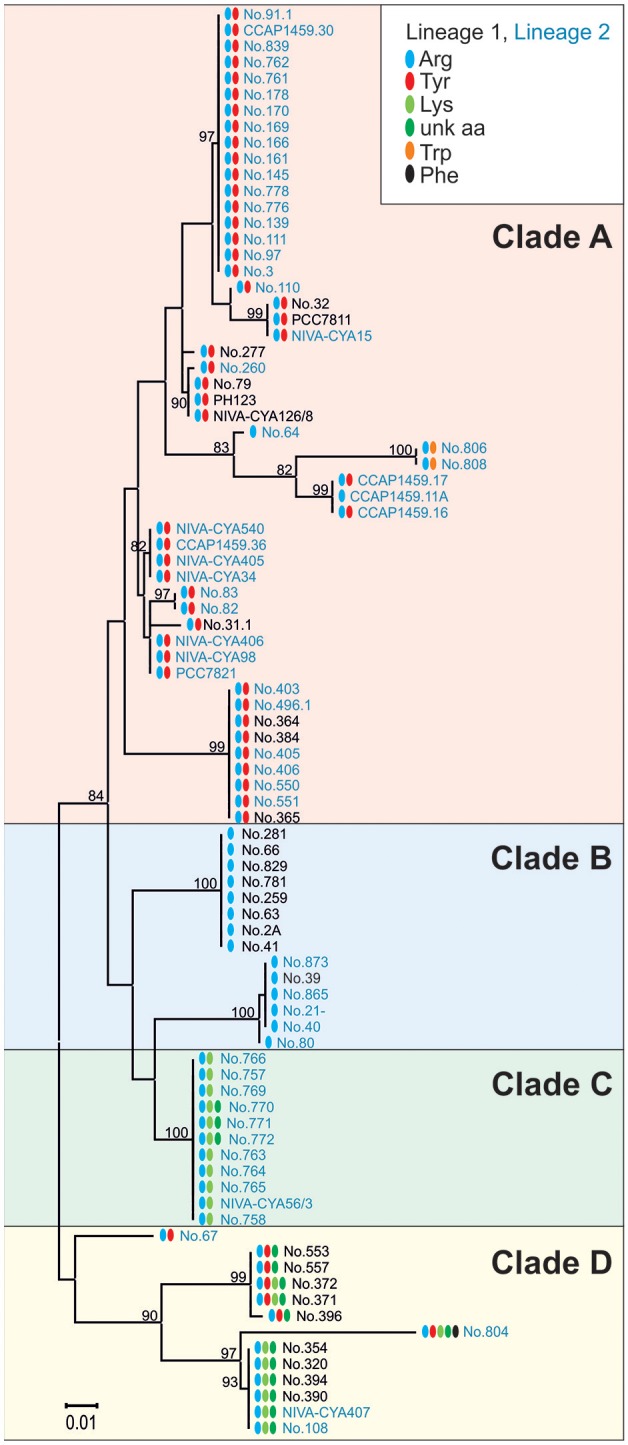
**Maximum likelihood tree of the ***apnA*** A_**1**_ sequences constituting the catalytic domain for substrate activation (core A4-A6)**. Numbers at the nodes indicate the percent bootstrap frequency (1,000 replicates). Strains of Lineage 1 are colored black, while strains of Lineage 2 are colored blue. Amino acid variability in pos. 1 of the AP molecule is indicated (Arg, blue; Tyr, red; Trp, orange; Lys, lime; unknown aa, green; Phe, black).

To determine the functional consequences of the genetic variability in the *apnA* A_1_ domain, sequences of all the AP-producing strains were compared with the structural variation occurring at the exocyclic position 1 of the AP molecule. In total, the maximum nucleotide variability among *apnA* A_1_-genotypes was 10.7% (*n* = 89). Phylogenetic analysis of the catalytic region, including the core motifs A4–A6 (510 bp), revealed four clades which corresponded with substrate activation in pos. 1 of the AP molecule (Figure 6). Clade A contained strains typically co-producing AP structural variants with Arg and Tyr in exocyclic pos. 1. As an exception, two strains (no. 806, 808) produced AP variants with Arg and Trp in this position, but they showed a slightly different *apnA* A_1_ sequence compared to the others. Strains among clade B carried only Arg at the exocyclic position, while strains among clade C produced Arg, Lys, and eventually an unknown aa. Clade D contained strains with a variety of AP structural variants with different amino acids in exocyclic pos. 1, either only Arg and Tyr, or combinations of Arg and/or Tyr and/or Lys and/or an unknown aa. Within clade D strain no. 804 co-produced even five AP variants differing in pos. 1 (Arg, Tyr, Lys, Phe, and an unknown aa).

Non-synonymous polymorphism within the binding site (core motif A4 and A6) resulted in 21 genotypes (a-u, max. 13.8% aa variability). Notably, different combinations of point substitutions were correlated to the same amino acid substrate activation across all phylogenetic Lineages 1 (1A) and 2 (2A), (Table 2). The bioinformatic predictions of A-domain selectivity (Rausch et al., 2005) resulted in 12 different specificity-conferring codes (Table 2). In addition to the ApnA A_1_-genotypes (a-c) related to Arg only in exocyclic pos. 1 of the AP molecule, ApnA A_1_-genotypes (i-t) were related to the co-occurrence of AP structural variants carrying Arg and Tyr in pos. 1 as described previously (Christiansen et al., 2011). Another group of ApnA A_1_-genotypes (f-h) was related to the co-occurrence of three amino acids Arg, Tyr, Lys in exocyclic pos. 1 of the AP molecule. As part of the specificity-conferring code, pos. 322 (Stachelhaus et al., 1999), located in the core motif A5 (NxYGPTE), is known to play a role in substrate selection (Stachelhaus et al., 1999; Challis et al., 2000). In *Planktothrix* strains, we found a substitution of Ala or Val vs. Gly at this position (pos. 298 of ApnA in reference genome NIVA-CYA 126/8).

**Table 2:** **ApnA A_**1**_ genotypes (core motif A4–A6) and specificity-conferring codes according to Stachelhaus et al. (1999)**.

**Genotypeamino acid level**	**Example strain**	**N**	**Distribution in lineages**				**Specifity-conferring code**	**% Similarity nucleotide level**	**Incorporated amino acid in pos. 1 (determined by HPLC-MS/MS)**					
			**1**	**1A**	**2**	**2A**			**Arg**	**Tyr**	**Lys**	**unk**.	**Phe**	**Trp**
a	No. 66^*^	8	8				DVEDIGAIEK	100	+					
b	No. 39^*^, No. 80^*^	6	1		3	2	DVEDIGAVEK	99.8–100	+					
c	No. 64	1			1		DVESIGAIEK	–	+					
d	No. 770	11				11	DVEDIGGVEK	100	+		+	(+)		
e	No. 108	6	3	1	2		DVEDIGGIEK	100	+		+	+		
f	No. 804	1			1		DMEDIGGIEK	–	+	+	+	+	+	
g	No. 371	4		4			DVESIGGIAK	100	+	+	(+)	+		
h	No. 396	1		1			DVESIGGIAK	–	+	+	+	+		
i	No. 67	1			1		DVEDIGIIAK	–	+	+				
j	No. 365	9		3	6		DVESIGAIAK	100	+	+				
k	No. 277	1		1			DVESIGAIAK	–	+	+				
l	NIVA-CYA 126/8^*^	3	3				DVESIGAIAK	100	+	+				
m	No. 260	1			1		DVESIGAIAK	–	+	+				
n	No. 32	3	2			1	DVESIGAIAK	100	+	+				
o	No. 3^*^	18			18		DVESIGAIAK	99.2–100	+	+				
p	No. 82	2			2		DVESIGVIAK	100	+	+				
q	PCC 7821^*^	3			3		DVESIGVIAK	100	+	+				
r	No. 31/1	1	1				DVESIGVIAK	–	+	+				
s	CCAP 1459.36^*^	4			1	3	DVESIGVIAK	100	+	+				
t	CCAP 1459/17	3			3		DVHSIGAIEK	100	+	(+)				
u	No. 806	2			2		DVHDIGAIEK	100	+					+

* *From Christiansen et al. (2011)*.

*+, AA detected in pos. 1 of AP molecule from all strains, (+), AA detected in pos. 1 of AP molecule from some strains*.

The ratio of non-synonymous (dN) and synonymous (dS) *apnA* A_1_ substitution rates per codon site for the entire phylogenetic tree (Figure 6) was dN/dS = 0.22. Site models revealed a small number of positively selected sites (W277I/L, S278D, A322/V/I/G, and E331A, numbering following Stachelhaus et al., 1999), (One ratio site model, M7 vs. M8, 2Δl = 6.32, *df* = 2, *p* < 0.05; Supplemental Figure S9). These sites were partly found critical for the bispecificity of the A-domain by causing a conformational change (Kaljunen et al., 2015; Ser243, Ala307).

In summary, the close match between bioinformatic prediction, dN/dS site model prediction, and comparison with crystallographic analysis implies that the evolution of *apnA* A_1_ genotypes occurred toward increased structural diversity through a small number of positively selected point mutations and independently from phylogenetic association.

## Discussion

### Evolution of the anabaenopeptin peptide synthesis gene cluster

The *apnA-E* genes from *Planktothrix* were notably found highly similar to the corresponding *apnA-E* genes from *Microcystis* strains when compared to *apnA-E* gene orthologues of *Anabaena, Nodularia, Nostoc* (Figure 4A). As opposed to microcystin synthesis (*mcy*) genes, the high similarity within *Microcystis* and *Planktothrix* among *apnA-E* genes and gene operon structure supports the hypothesis of a HGT event. Furthermore, even the flanking region at the 5′end of the *apnA-E* gene cluster (4,370 bp) was found to be identical between *Planktothrix* and *Microcystis* PCC 9432. As both genera share a planktonic lifestyle and frequently co-occur in high numbers in shallow polymictic habitats of the temperate climatic zone, such HGT events appear reasonable. In addition, no evidence for *apnA-E* gene cluster remnants in the more distantly related strains *P. mougeotii* or *P. tepida* assigned to Lineage 3 were found (Figure 2). In contrast, all of the strains of Lineage 1 lacking the *apnA-E* gene cluster (i.e., PCC 7805) carried remnants of the *apnA-E* gene cluster 5′end flanking region. It is thus concluded that strains of Lineage 1 showing this flanking region actually lost the *apnA-E* gene cluster. Similar to the loss process of microcystin synthesis in *Planktothrix* strains (Christiansen et al., 2008), this *apn* gene cluster loss event did not lead to phylogenetic separation but rather strains carrying or lacking the *apn* genes co-occurred in the same habitat (i.e., strains no. 263, 274 vs. no. 259, 281 isolated from Lake Wannsee, Berlin, Germany).

Recently, Calteau et al. (2014) compared 126 genomes of different cyanobacteria strains and discovered a patchy distribution of various NRPS/polyketide synthase (PKS) gene clusters throughout the whole cyanobacteria phylum. The authors concluded that the history of different NRPS gene clusters can differ and while some of these gene clusters seemed to be transmitted through HGT (as inferred from mobile flanking regions or nucleotide composition), others revealed complex vertical gene cluster evolution (e.g., gene duplication, recombination, inversion, and gene loss). It might be hypothesized that within a specific genus, such as *Planktothrix*, the history and relative age of various peptide synthesis gene clusters also differ. A hypothetical early ancestor of the genus *Planktothrix* is unlikely to contain all the different NRPS gene clusters, which would lead to the co-occurrence of at least 9 different NRPS or PKS or NRPS/PKS hybrid or RiPP gene clusters (microcystin, AP, cyanopeptolin, aeruginosin, oscillaginin, microviridin, prenylagaramide, planktocyclin, and luminaolide, e.g., see Ueoka et al., 2015; Kurmayer et al., 2016). Thus, the probability that only vertically inherited gene loss processes resulted in the today's secondary metabolite structural diversity is low. In contrast to vertical inheritance, we hypothesize that the *apnA-E* gene cluster was introduced into the chromosome of the ancestor of Lineage 1 (*P. agardhii*) by HGT and became phylogenetically fixed among the strains of Lineage 2 (Figure 2). As the MLSA suggests a more recent evolution of Lineage 2 out of Lineage 1 (Kurmayer et al., 2015), a genotype carrying the *apn* gene cluster was the hypothetical ancestor of Lineage 2. The same conclusion has been obtained for microcystin synthesis previously (Christiansen et al., 2008). It has been hypothesized by Suda et al. (2002) that red-pigmented Lineage 2 is evolutionary relatively young and, therefore, shows a higher similarity in nucleotide sequences as revealed by DNA-DNA hybridization. In contrast, the phylogenetic tree revealed by MLSA (Figure 2) did not reveal lower genetic divergence among the strains of Lineage 2 when compared with the diversity among the strains of Lineage 1. Thus, the absolute presence of microcystin and anabaenopeptin among the strains of Lineage 2 is striking and the question arises here as to whether the *apn* gene cluster presence is linked to the ecological diversification of Lineage 2. The first results from the comparison of the average nucleotide identity (ANI) for all of the strains with sequenced genome of Lineage 1 and 2 (Table 1) revealed high similarity within phylogenetic lineages (98.9–100% in Lineage 1 vs. 98.1–100% in Lineage 2) but lower similarity in between them (96.9–98%). In contrast, the nucleotide variability of *apn* genes is up to 14% among strains of Lineage 1 (no. 66, no. 365, NIVA-CYA 126/8) and only 2% among the strains of Lineage 2 (no. 758, no. 82, PCC 7821, no. 108). Thus, the 100% occurrence of the *apnA-E* gene cluster combined with the low variability of *apn* genes among Lineage 2 might indeed imply purifying selection.

### Functional consequences of A-Domain nucleotide variability and structural diversity

For the different peptide families produced by *Planktothrix*, structural variants with significant toxicity/bioactivity have been reported (e.g., Kurmayer et al., 2016). Thus, a common function of the different resulting products has been suggested, such as chemical defense through the inhibition of eukaryotic protein phosphatases 1 and 2A (microcystins, APs) and different proteases including metallocarboxypeptidases (APs) or serine proteases (cyanopeptolins, microviridins). In general, the high intracellular concentrations preclude extracellular functions but rather deter parasites such as chytrid fungi (Rohrlack et al., 2013), amoeba (Dirren et al., 2014), and not at least herbivorous crustaceans (Blom et al., 2006). It has been argued that microcystin and other bioactive peptides cannot inhibit the feeding activity of crustaceans, for example when using microcystin-deficient experimental mutants when compared to the wild type (Rohrlack et al., 1999). Under natural conditions, cyanobacteria grow in macroscopic colonies/filaments and predators, such as herbivorous copepods, typically feed by biting off portions of filaments that are not totally consumed (the so-called filament clipping (Schaffner et al., 1994). In addition, protozoans were fed by engulfing the tip of a filament (Dirren et al., 2014) or by breaking of filaments (e.g., the ciliate *Obertrumia aurea*; Posch et al., 2012). This feeding behavior on large macroscopic *Planktothrix* is comparable to the grazing behavior by insects on higher plants resulting in the intracellular production of protease inhibitors either found constitutively in various parts of the plant or which may be induced in response to mechanical attack (e.g., Jongsma and Bolter, 1997). Thus, individuals with a more effective chemical defense will survive and the combination of different protease inhibitors is generally considered advantageous to the producer to combat co-evolutionary responses. Intercellular cocktails of toxins/bioactive peptides may provide a better protection due to synergistic interactions but also due to the impediment of a co-evolutionary response in aquatic predators (Schwarzenberger and Von Elert, 2013). In this study, except for the strain CCAP 1459/31, all the strains carrying the *apnA-E* gene cluster produced at least one AP structural variant. Since all AP producers contained AP carrying Arg in pos. 1, we think that this structural AP variant is the original one. Notably, high inhibitory activity of AP variants with Arg in exocyclic pos. 1 has been reported for carboxypeptidase B, i.e., the activity drops in the order Arg = Lys > Tyr > Phe ≈ Ile (Schreuder et al., 2016). For carboxypeptidase A rather a reverse order of potency has been reported, with aromatic/aliphatic aa in exocyclic pos. 1 showing high activity and Arg with lower activity (references in Schreuder et al., 2016). Thus, it might be that natural enzymatic targets of the most abundant AP B and AP F structural variants resemble more carboxypeptidase B than the frequently tested carboxypeptidase A. Nevertheless, single nucleotide polymorphism led to the co-synthesis of AP structural variants carrying either Arg/Tyr (48 strains), Arg/Lys (8 strains), Arg/Trp (2 strains), or other aa combinations in pos. 1 of the AP molecule. From crystallographic analysis, it is known that ApnA A_1_-domain genotypes carrying substitutions pos. 278 and pos. 331 (numbering following Stachelhaus et al., 1999) led to conformational change allowing for Arg and Tyr activation (Kaljunen et al., 2015). It remains to be shown whether Arg/Lys or Arg/Trp co-activation is enabled by a similar biochemical mechanism. Notably, even strains co-producing APs with three different amino acids (Arg/Tyr/Lys) in pos. 1 were identified. The occurrence of these potentially functional point mutations within *apnA* A_1_ domain does not relate to a specific phylogenetic lineage but is rather distributed across the entire phylogeny. The incongruence with phylogeny can be best explained by a regular exchange of shorter DNA fragments from the NRPS A-domains of different genotypes across phylogenetic lineages, as it was described earlier for similar enzymes of microcystin biosynthesis (Kurmayer and Gumpenberger, 2006). However, the high frequency of promiscuous ApnA A_1_-domain genotypes points to its ecological significance. In contrast, the recombination affecting the *apnC-D* genes, leading to the substitution of Ala vs. Hty and Phe vs. Ile in pos. 5, 6 of the AP molecule (Christiansen et al., 2011), was found much less frequent—only in three strains. Thus, this larger fragment recombination is considered selectively neutral. Accordingly, replacing all aa in pos. 1–6 in AP-type peptides (called brunsvicamides; isolated from *Tychonema* sp.) confirmed the essential role of D-Lys in pos. 2 and Ile in the exocyclic pos. 1 (Walther et al., 2009), but also showed that replacing aa in pos. 3–6 had no effect on carboxypeptidase A inhibitory activity. In summary, an evolutionary process leading to promiscuous ApnA A_1_-genotypes is plausible.

## Author contributions

EE acquired the data, performed data analysis and interpretation, and wrote parts of the present manuscript. MF and GC assisted in data acquisition, data analysis and data interpretation. LD assisted in manuscript writing. JB assisted in data analysis. RK designed the work, assisted in data analysis and interpretation, and the writing and submission of the present manuscript.

### Conflict of interest statement

The authors declare that the research was conducted in the absence of any commercial or financial relationships that could be construed as a potential conflict of interest.
