# Supplementary material for: Evolution of Anabaenopeptin Peptide Structural Variability in the Cyanobacterium Planktothrix
Source: Front Microbiol. 2017 Feb 16;8:219. doi: 10.3389/fmicb.2017.00219 (PMC5311044; doi:10.3389/fmicb.2017.00219)
Supplement: Supplementary file 1 [file DataSheet1.pdf]

Supplemental information

Supplementary Table S1. List of various anabaenopeptins found in the *Planktothrix* strains. Countries indicated according to two-letter code.

								AP structural variants[a]																	
Phylogenetic Lineage	Strain	Origin geographic location (Country code ISO-3166-1993)	Pigmentation	5'end flanking region (*apnA-ociD*)	*apnA-E*	3'end flanking region (*apnE-mvdA*)	*ociD-aspB*	Unk AP 1, 884.4 Da, 12.3 min	AP C, 808.4 Da, 15.5 min	Unk AP 2, 822.5 Da, 16.0 min	AP B, 836.5 Da, 16.4 min	Unk AP 3, 822.5 Da, 17.9 min	Osc B, 868.4 Da, 18.2 min	Unk AP 4, 836.5 Da, 18.4 min	AP 908, 908.5 Da, 18.4 min	AP F, 850.5 Da, 18.8 min	Unk AP 5, 822.5 Da, 19.1 min	Unk AP 6, 850.5 Da, 19.6 min	Unk AP 7, 850.5 Da, 20.0 min	AP A, 843.4 Da, 21.9 min	AP 915, 915.5 Da, 23.9 min	Osc Y, 857.4 Da, 24.6 min	AP D, 827.4 Da, 29.1 min	FA A, 866.4 Da, 29.2 min	Unk AP 8, 841.4 Da, 32.2 min
1	No. 2A	L. Markusbölefjärden (FI)	G	Y	Y	nd	na				X														
1	No. 31/1	Wannsee (DE)	G	Y	Y	nd	na				x					X				x		X			
1	No. 32	-´´-	G	Y	Y	Y	na				X									X					
1	No. 39	-´´-	G	Y	Y	nd	na				X					X									
1	No. 41	Jägerteich (AT)	G	Y[b]	Y	nd	na				X														
1	No. 63	-´´-	G	Y[b]	Y	nd	na				X														
1	No. 66	-´´-	G	Y[b]	Y	nd	na				X														
1	No. 79	L. Arresø (DK)	G	Y	Y	Y	na				X									X					
1	No. 250	Albufera Lagune (ES)	G	nd	nd	na	Y																		
1	No. 251	-´´-	G	nd	nd	na	Y																		
1	No. 252	-´´-	G	nd	nd	na	Y																		
1	No. 253	-´´-	G	nd	nd	na	Y																		
1	No. 254	-´´-	G	nd	nd	na	Y																		
1	No. 255	-´´-	G	nd	nd	na	Y																		

1	No. 256	-´´-	G	nd	nd	na	Y														
1	No. 257	-´´-	G	nd	nd	na	Y														
1	No. 259	Wannsee (DE)	G	Y	Y[c]	nd	na							X							
1	No. 263/2	-´´-	G	nd	nd	na	Y														
1	No. 274	-´´-	G	nd	nd	na	Y														
1	No. 281	-´´-	G	Y	Y[c]	nd	na							X							
1	No. 299	Klinkenbergerplas (NL)	G	nd	nd	na	Y														
1	No. 307	-´´-	G	nd	nd	na	Y														
1	No. 320	-´´-	G	Y	Y	nd	na	X	x	X	X		X		X						
1	No. 354	Moose L. (CA)	G	Y	Y[d]	nd	na	X	x	X											
1	No. 394	-´´-	G	Y	Y[d]	nd	na	X	x	X						x		x			
1	No. 759	São Domingos (PT)	G	nd	nd	na	Y														
1	No. 760	-´´-	G	nd	nd	na	Y														
1	No. 781	L. Nero (RU)	G	Y	Y	nd	na			X								x			
1	No. 787	São Domingos (PT)	G	nd	nd	na	Y														
1	No. 788	-´´-	G	nd	nd	na	Y														
1	No. 790	-´´-	G	nd	nd	na	Y														
1	No. 828	L. Nero (RU)	G	nd	nd	na	Y														
1	No. 829	-´´-	G	Y	Y	nd	na			X											
1	No. 976	Ft. Lowell, Tucson (US)	G	nd	nd	na	Y														
1	CCAP 1459/15	Lough Neagh (UK)	G	nd	nd	na	Y														
1	NIES 596	Veluwermeer (NL)	G	nd	nd	na	Y														
1	NIVA-CYA 116	L. Arungen (NO)	G	nd	nd	na	Y														
1	NIVA-CYA 126/8	L. Langsjön (FI)	G	Y	Y[c]	Y	nd							X						X	
1	PH 123	Lake Lange Sø (DK)	G	Y	Y	Y	na			X									X		
1	PCC 7805	Veluwemeer (NL)	G	nd	nd	na	Y														
1	PCC 7811	Paris, Vert le Petit (FR)	G	Y	Y	Y	na								X						X
1	SAG 5.81	Kiessee (DE)	G	nd	nd	na	Y														
1A	No. 277	Wannsee (DE)	G	Y	Y	nd	na			X									X		
1A	No. 364	Moose L. (CA)	G	Y	Y[d]	nd	na			X									X		
1A	No. 365	-´´-	G	Y	Y[d]	nd	na			X									X		

1A	No. 371	-´´-	R	Y	Y	Y	na	x	X	X				X	
1A	No. 372	-´´-	R	Y	Y	Y	na	x	X	X				X	
1A	No. 384	-´´-	G	Y	Y[d]	nd	na			X				X	
1A	No. 390	-´´-	G	Y	Y[d]	nd	na	X	x	X					
1A	No. 396	-´´-	R	Y	Y	Y	na	x	x	X				X	
1A	No. 553	-´´-	R	Y	Y	Y	na		X	X				X	
1A	No. 557	-´´-	R	Y	Y	Y	na		X	X				X	
2	No. 3	Mondsee (AT)	R	Y	Y	Y	na			X			X	x	x
2	No. 21-	Figur (AT)	R	Y	Y	Y	na			X			X		
2	No. 40	Mondsee (AT)	R	Y	Y	Y	na			X			X		
2	No. 64	Wörthersee (AT)	R	Y	Y	Y	na			X			X		
2	No. 67	Wörthersee (AT)	R	Y	Y	Y	na			X				X	
2	No. 80	Schwarzensee (AT)	R	Y	Y	Y	na			X					
2	No. 82	Ammersee (DE)	R	Y	Y	Y	na			x			X	x	X
2	No. 83/2	-´´-	R	Y	Y	Y	na			x			X	X	X
2	No. 91/1	Mondsee (AT)	R	Y	Y	Y	na			X			X	X	X
2	No. 97	-´´-	R	Y	Y	Y	na			X			X	X	x
2	No. 108	Irrsee (AT)	R	Y	Y	Y	na	x		x	X	x	X		
2	No. 110	Mondsee (AT)	R	Y	Y	Y	na			X			X	x	x
2	No. 111	-´´-	R	Y	Y	Y	na			X			X	X	x
2	No. 139	Grabensee (AT)	R	Y	Y	Y	na			X			X	X	X
2	No. 145	-´´-	R	Y	Y	Y	na			X			X	X	X
2	No. 161	-´´-	R	Y	Y	Y	na			X			X	X	X
2	No. 166	-´´-	R	Y	Y	Y	na			x			X	x	X
2	No. 169	-´´-	R	Y	Y	Y	na			X			X	X	X
2	No. 170	-´´-	R	Y	Y	Y	na			x			X	x	X
2	No. 178	-´´-	R	Y	Y	Y	na			x			X	x	X
2	No. 260	Wannsee (DE)	G	Y	Y	Y	na			X				X	
2	No. 403	Moore(Crane) L. (CA)	R	Y	Y	Y	na			X				X	
2	No. 405	-´´-	R	Y	Y	Y	na			X				X	
2	No. 406	-´´-	R	Y	Y	Y	na			X				X	

2	No. 496/1	-´´-	R	Y	Y	Y	na				X									X					
2	No. 550	-´´-	R	Y	Y	Y	na				X									X					
2	No. 551	-´´-	R	Y	Y	Y	na				X									X					
2	No. 761	R. Garcia (IT)	R	Y	Y	Y	na				X					X				X		X			
2	No. 762	-´´-	R	Y	Y	Y	na				X					X				X		X			
2	No. 776	R. Nicolletti (IT)	R	Y	Y	Y	na				x					X				x		X			
2	No. 778	-´´-	R	Y	Y	Y	na				X					X				x		X			
2	No. 804	Winnecook L. (US)	G	Y	Y	Y	na		X	x	x						x			x			X		x
2	No. 806	-´´-	G	Y	Y	Y	na				x													X	
2	No. 808	-´´-	G	Y	Y	Y	na				X													X	
2	No. 839	R. Garcia (IT)	R	Y	Y	Y	na				x					X				x		X			
2	CCAP 1459/11A	L. Windermere (UK)	G	Y	Y	Y	na	x					X					x							
2	CCAP 1459/16	Blelham Tarn (UK)	G	Y	Y	Y	na				X									x					
2	CCAP 1459/17	-´´-	G	Y	Y	Y	na				X									X					
2	CCAP 1459/30	Plöner See (DE)	R	Y	Y	Y	na									X						X			
2	CCAP 1459/31	White Lough (IR)	G	Y	Y^e	Y	na																		
2	NIVA-CYA 98	L. Steinsfjorden (NO)	R	Y	Y	Y	na				x					X						X			
2	NIVA-CYA 406	-´´-	R	Y	Y	Y	na				x					X						X			
2	NIVA-CYA 407	-´´-	R	Y	Y	Y	na				x	X		x		X									
2	NIVA-CYA 540	-´´-	R	Y	Y	Y	na				X					X				X		X			
2	PCC 7821	L. Gjersjoen (NO)	R	Y	Y	Y	na				x					X				x		X			
2A	No. 757	L. Hormajärvi (FI)	G	Y	Y	Y	na		X		X														
2A	No. 758	-´´-	G	Y	Y	Y	na		X		X														
2A	No. 763	-´´-	G	Y	Y	Y	na		X		X														
2A	No. 764	-´´-	G	Y	Y	Y	na		X		X														
2A	No. 765	-´´-	G	Y	Y	Y	na		X		X														
2A	No. 766	-´´-	G	Y	Y	Y	na		X		X														
2A	No. 769	-´´-	G	Y	Y	Y	na		X		X														
2A	No. 770	-´´-	G	Y	Y	Y	na		X	x	X														
2A	No. 771	-´´-	G	Y	Y	Y	na		X	x	X														
2A	No. 772	-´´-	G	Y	Y	Y	na		X	x	X														

2A	No. 865	L. Hiidenvesi (FI)	G	Y	Y	Y	na		x		X		
2A	No. 873	-´´-	G	Y	Y	Y	na		x		X		
2A	CCAP 1459/36	L. Gjersjoen (NO)	G	Y	Y	Y	na		X		X	X	X
2A	NIVA-CYA 15	L. Kolbotnvatnet (NO)	G	Y	Y	Y	na		X			X	
2A	NIVA-CYA 34	-´´-	G	Y	Y	Y	na		x		X	X	X
2A	NIVA-CYA 56/3	L. Steinsfjorden (NO)	G	Y	Y	Y	na	x	X	X	X		
2A	NIVA-CYA 405	-´´-	G	Y	Y	Y	na		X		X	X	X
3	No. 704	Saka (UG)	G	nd	nd	na	nd						
3	No. 707	-´´-	G	nd	nd	na	na						
3	No. 708	-´´-	G	nd	nd	na	nd						
3	No. 710	-´´-	G	nd	nd	na	na						
3	No. 711	-´´-	G	nd	nd	na	na						
3	No. 712	-´´-	G	nd	nd	na	nd						
3	No. 713	-´´-	G	nd	nd	na	na						
3	No. 980	Ft. Lowell, Tucson (US)	G	nd	nd	na	na						
3	No. 1020	Vaal River (ZA)	G	nd	nd	na	na						
3	CCAP 1460/6	Nakhon Pathon (TH)	G	nd	nd	na	na						
3	PCC 9214	Landjia (CF)	G	nd	nd	na	na						

[a] *sensu* Harada et al. 1995 (AP B $[M+H]^+$ 837.5, AP A $[M+H]^+$ 844.4), Shin et al. 1997 (AP F $[M+H]^+$ 851.5), Sano and Kaya 1995 (Osc Y $[M+H]^+$ 858.4), Fujii et al. 2002 (AP C $[M+H]^+$ 809.4, AP D $[M+H]^+$ 828.4), Okumura et al. 2009 (AP 908 $[M+H]^+$ 909.5, AP 915 $[M+H]^+$ 916.5), Sano et al. 2001 (Osc B $[M+H]^+$ 869.4), Williams et al. 1996 (ferintoic acid A $[M+H]^+$ 867.4)

[b] Insertion IS1634; [c] Recombination within *apn*C and *apn*D; [d] Recombination within *apn*E; [e] Partial deletion within *apn*B

R, red; G, green; Y, yes; nd, not detected; na, not analyzed; X, major peptide (30-100% peak area compared to the most abundant AP); x, minor peptide (5-30% peak area compared to the most abundant AP)

Supplementary Table S2. Primer pairs used to amplify the *apnA-E* gene cluster and its flanking regions from *Planktothrix* strains.

Region	Primer name	Primer sequence 5'-3'[1]	T_A [°C]	Amplicon size [bp]	Elongation time [min]
*apn*A-E gene cluster	apn1F+<br>apn1F-	CCTCCATGTTCAAGCAATCT<br>CGGAATATTTCATGACGACG	60	2,017	1
	apn2+<br>apn2-	TATCAGTGAAATTCTACGTCGTCATG<br>TGAGACAGATTTCATCATTTCTTCTG	60	1,975	1
	apn3+<br>apn3-	CAGAAGAAATGATGAAATCTGTCTCA<br>CCAACCAACAGTACGAGACAAGT	60	2,024	1
	apn4+<br>apn4-	ACTTGTCTCGTACTGTTGGTTGG<br>CGATCGCATTAGGAGTCTTTTCTA	60	2,027	1
	apn5+<br>apn5-	TAGAAAAGACTCCTAATGCGATCG<br>GTTGTGCCACTTCATCTTCAGATAA	60	1,800	1
	apn6+<br>apn6-	TTATCTGAAGATGAAGTGGCACAAC<br>TCTTCTGGATAATCGGAGTCAATC	60	1,962	1
	apn7+<br>apn7Tres-	GATTGACTCCGATTATCCAGAAGA<br>GCACGATAAAGTTGTTGCCATT	60	2,007	1
	apn8Tres+<br>apn8Tres-	AATGGCAACAACTTTATCGTGC<br>GGGATGATTCCAATAACCTTGAG	60	2,009	1
	apn9Tres+<br>apn9-	CTCAAGGTTATTGGAATCATCCC<br>CGTAACTTGTCTCCATCAGCAGA	60	2,079	1
	apn10+<br>apn10Tres-	TCTGCTGATGGAGACAAGTTACG<br>GAAATGACTCAAAATTAAGCCGATC	60	1,951	1
	apn11TRes+<br>apn11-	GATCGGCTTAATTTTGAGTCATTTC<br>TGGGTTGGTAATCCGCATAAT	60	1,972	1
	apn12+<br>apn12-	ATTATGCGGATTACCAACCCA<br>CTGATAGTGCCTATTGAGTAGTTGGTC	60	2,287	1

*apn*CD_recombination	apn7+<br>apn7T126-	GATTGACTCCGATTATCCAGAAGA<br>AGGCATTATAAGCTTGTTCCCATT	60	2,009	1
	apn8T126+<br>apn8T126-	AATGGGAACAAGCTTATAATGCCT<br>TTCGCCATTTAGCTAAGTCTCCT	60	2,083	1
	apn9T126+<br>apn9-	AGGAGACTTAGCTAAATGGCGAA<br>CGTAACTTGTCTCCATCAGCAGA	60	2,003	1
	apn10+<br>apn10T126-	TCTGCTGATGGAGACAAGTTACG<br>TCAAGGTTCTTGCCTTGAGATAAC	60	1,910	1
	apn11T126+<br>apn11-	GTTATCTCAAGGCAAGAACCTTGA<br>TGGGTTGGTAATCCGCATAAT	60	2,036	1
No *apn* gene cluster	ClbrCptD-<br>AspB-	AGCAACTATTCCGTCAGTAGT<br>CTACTCGCGCTGCTAGCTT	58	5,073	2.5
5'end flanking region	ClbrCptD-<br>ClBrApnA+	AGCAACTATTCCGTCAGTAGT<br>TTAATTCCTCATAGGTCAATTG	60	4,822	2
3'end flanking region	Apn12+<br>ClBrMvdA+	ATTATGCGGATTACCAACCCA<br>TTATAGGTTTCTTCGTCCAT	60	4,376	2
*apn*A A_1 domain	apn1F+<br>apnAA1a-	CCTCCATGTTCAAGCAATCT<br>CGAGATAAAATTTGTGTGGCAAT	60	1,702	1

[1] Based on the *apnA-E* gene clusters obtained from genome sequencing of strains NIVA-CYA 98 (access. no. AM990463.2) and NIVA-CYA 126/8 (access. no. CM002803.1).

Supplementary Table S3. AP structural variants detected in *Planktothrix* strains.

	Da	RT [min]	Pos. 1	Pos. 2	Pos. 3	Pos. 4	Pos. 5	Pos. 6
Unknown AP 1	884.4	12.3	Arg	Lys	165 Da*	Hty	N-Me-Ala	Phe
Anabaenopeptin C	808.4	15.5	Lys	Lys	Val	Hty	N-Me-Ala	Phe
Unknown AP 2	822.5	16.0	160 Da*	Lys	Val	Hty	N-Me-Ala	Phe
Anabaenopeptin B	836.5	16.4	Arg	Lys	Val	Hty	N-Me-Ala	Phe
Unknown AP 3	822.5	17.9	Lys	Lys	Ile	Hty	N-Me-Ala	Phe
Oscillamide B	868.4	18.2	Arg	Lys	Met	Hty	N-Me-Ala	Phe
Unknown AP 4	836.5	18.4	160 Da*	Lys	Ile	Hty	N-Me-Ala	Phe
Anabaenopeptin 908	908.5	18.6	Arg	Lys	Ile	Hty	N-Me-Hty	Ile
Anabaenopeptin F	850.5	18.8	Arg	Lys	Ile	Hty	N-Me-Ala	Phe
Unknown AP 5	822.5	19.1	Lys	Lys	Val	209 Da*	N-Me-Ala	Phe
Unknown AP 6	850.5	19.6	Arg	Lys	117 Da*	Hty	N-Me-Ala	Phe
Unknown AP 7	850.5	20.0	Arg	Lys	Val	209 Da*	N-Me-Ala	Phe
Anabaenopeptin A	843.4	21.9	Tyr	Lys	Val	Hty	N-Me-Ala	Phe
Anabaenopeptin 915	915.5	23.9	Tyr	Lys	Ile	Hty	N-Me-Hty	Ile
Oscillamide Y	857.4	24.6	Tyr	Lys	Ile	Hty	N-Me-Ala	Phe
Anabaenopeptin D	827.4	29.1	Phe	Lys	Val	Hty	N-Me-Ala	Phe
Ferintoic Acid A	866.4	29.2	Trp	Lys	Val	Hty	N-Me-Ala	Phe
Unknown AP 8	841.4	32.2	Phe	Lys	Val	209 Da*	N-Me-Ala	Phe

* Molecular mass of the free amino acid

Supplementary Table S4. MS fragments of different AP structural variants detected in *Planktothrix* strains.

Anabaenopeptin B		Anabaenopeptin A		Anabaenopeptin C	
m/z	Sequence	m/z	Sequence	m/z	Sequence
837.5	M + H	844.5	M + H	809.5	M + H
637.4	Lys + Val + Hty + MAla + Phe + 2H	637.4	Lys + Val + Hty + MAla + Phe + 2H	637.4	Lys + Val + Hty + MAla + Phe + 2H
619.4	Lys + Val + Hty - H_2O + MAla + Phe + H	619.4	Lys + Val + Hty - H_2O + MAla + Phe + H	619.4	Lys + Val + Hty - H_2O + MAla + Phe + H
534.3	Lys + Val + Hty - H_2O + Phe + H	534.3	Lys + Val + Hty - H_2O + Phe + H	534.3	Lys + Val + Hty - H_2O + Phe + H
460.3	Lys + Val + MAla + Phe + 2H	460.3	Lys + Val + MAla + Phe + 2H	460.3	Lys + Val + MAla + Phe + 2H
387.2	Lys + Val + Hty - H_2O + H	387.2	Lys + Val + Hty - H_2O + H	387.2	Lys + Val + Hty - H_2O + H
370.2	Lys + Val + Hty - H_2O - NH_2 + H	370.2	Lys + Val + Hty - H_2O - NH_2 + H	370.2	Lys + Val + Hty - H_2O - NH_2 + H
Unknown Anabaenopeptin 2		Anabaenopeptin D		Ferintoic Acid A	
m/z	Sequence	m/z	Sequence	m/z	Sequence
823.5	M + H	828.5	M + H	867.5	M + H
637.4	Lys + Val + Hty + MAla + Phe + 2H	637.4	Lys + Val + Hty + MAla + Phe + 2H	637.4	Lys + Val + Hty + MAla + Phe + 2H
619.4	Lys + Val + Hty - H_2O + MAla + Phe + H	619.4	Lys + Val + Hty - H_2O + MAla + Phe + H	619.4	Lys + Val + Hty - H_2O + MAla + Phe + H
534.3	Lys + Val + Hty - H_2O + Phe + H	534.3	Lys + Val + Hty - H_2O + Phe + H	534.3	Lys + Val + Hty - H_2O + Phe + H
460.3	Lys + Val + MAla + Phe + 2H	460.3	Lys + Val + MAla + Phe + 2H	460.3	Lys + Val + MAla + Phe + 2H
387.2	Lys + Val + Hty - H_2O + H	387.2	Lys + Val + Hty - H_2O + H	387.2	Lys + Val + Hty - H_2O + H
Anabaenopeptin F		Oscillamide Y		Unknown Anabaenopeptin 3	
m/z	Sequence	m/z	Sequence	m/z	Sequence
851.6	M + H	858.5	M + H	823.5	M + H
651.4	Lys + Ile + Hty + MAla + Phe + 2H	651.4	Lys + Ile + Hty + MAla + Phe + 2H	651.4	Lys + Ile + Hty + MAla + Phe + 2H
633.4	Lys + Ile + Hty - H_2O + MAla + Phe + H	633.4	Lys + Ile + Hty - H_2O + MAla + Phe + H	633.4	Lys + Ile + Hty - H_2O + MAla + Phe + H
548.3	Lys + Ile + Hty - H_2O + Phe + H	548.3	Lys + Ile + Hty - H_2O + Phe + H	548.3	Lys + Ile + Hty - H_2O + Phe + H
474.3	Lys + Ile + MAla + Phe + 2H	474.3	Lys + Ile + MAla + Phe + 2H	474.3	Lys + Ile + MAla + Phe + 2H
401.2	Lys + Ile + Hty - H_2O + H	401.2	Lys + Ile + Hty - H_2O + H	401.2	Lys + Ile + Hty - H_2O + H

Unknown Anabaenopeptin 4		Unknown Anabaenopeptin 5		Unknown Anabaenopeptin 7	
m/z	Sequence	m/z	Sequence	m/z	Sequence
837.5	M + H	823.5	M + H	851.5	M + H
651.4	Lys + Ile + Hty + MAla + Phe + 2H	651.4	Lys + Val + (191 Da) + MAla + Phe + 2H	651.4	Lys + Val + (191 Da) + MAla + Phe + 2H
633.4	Lys + Ile + Hty - H_2O + MAla + Phe + H	633.4	Lys + Val + (191 Da) - H_2O + MAla + Phe + H	633.4	Lys + Val + (191 Da) - H_2O + MAla + Phe + H
548.3	Lys + Ile + Hty - H_2O + Phe + H	548.3	Lys + Val + (191 Da) - H_2O + Phe + H	548.3	Lys + Val + (191 Da) - H_2O + Phe + H
474.3	Lys + Ile + MAla + Phe + 2H	460.3	Lys + Val + MAla + Phe + 2H	460.3	Lys + Val + MAla + Phe + 2H
401.2	Lys + Ile + Hty - H_2O + H	401.2	Lys + Val + (191 Da) - H_2O + H	401.2	Lys + Val + (191 Da) - H_2O + H

Unknown Anabaenopeptin 8		Unknown Anabaenopeptin 6		Anabaenopeptin 908	
m/z	Sequence	m/z	Sequence	m/z	Sequence
842.5	M + H	851.5	M + H	909.6	M + H
651.4	Lys + Val + (191 Da) + MAla + Phe + 2H	651.4	Lys + (113 Da) + Hty + MAla + Phe + 2H	709.5	Lys + Val + Hty + MHty + Ile + 2H
633.4	Lys + Val + (191 Da) - H_2O + MAla + Phe + H	633.4	Lys + (113 Da) + Hty - H_2O + MAla + Phe + H	691.4	Lys + Val + Hty - H_2O + MHty + Ile + H
548.3	Lys + Val + (191 Da) - H_2O + Phe + H	548.3	Lys + (113 Da) + Hty - H_2O + Phe + H	518.4	Lys + Val + Hty + Ile + 2H
460.3	Lys + Val + MAla + Phe + 2H	474.3	Lys + (113 Da) + MAla + Phe + 2H	500.3	Lys + Val + MHty - H_2O + Ile + H
401.2	Lys + Val + (191 Da) - H_2O + H	401.2	Lys + (113 Da) + Hty - H_2O + H	387.2	Lys + Val + Hty - H_2O + H

Anabaenopeptin 915		Oscillamide B		Unknown Anabaenopeptin 1	
m/z	Sequence	m/z	Sequence	m/z	Sequence
916.5	M + H	869.4	M + H	885.4	M + H
709.5	Lys + Val + Hty + MHty + Ile + 2H	669.4	Lys + Met + Hty + MAla + Phe + 2H	685.4	Lys + (147 Da) + Hty + MAla + Phe + 2H
691.4	Lys + Val + Hty - H_2O + MHty + Ile + H	651.4	Lys + Met + Hty - H_2O + MAla + Phe + H	667.4	Lys + (147 Da) + Hty - H_2O + MAla + Phe + H
518.4	Lys + Val + Hty + Ile + 2H	566.3	Lys + Met + Hty - H_2O + Phe + H	621.4	Lys + (147 Da) – SCH_3+ Hty - H_2O + MAla + Phe + H
500.3	Lys + Val + MHty - H_2O + Ile + H	492.3	Lys + Met + MAla + Phe + 2H	508.1	Lys + (147 Da) + MAla + Phe + 2H
387.2	Lys + Val + Hty - H_2O + H	419.2	Lys + Met + Hty - H_2O + H	434.9	Lys + (147 Da) + Hty - H_2O + H

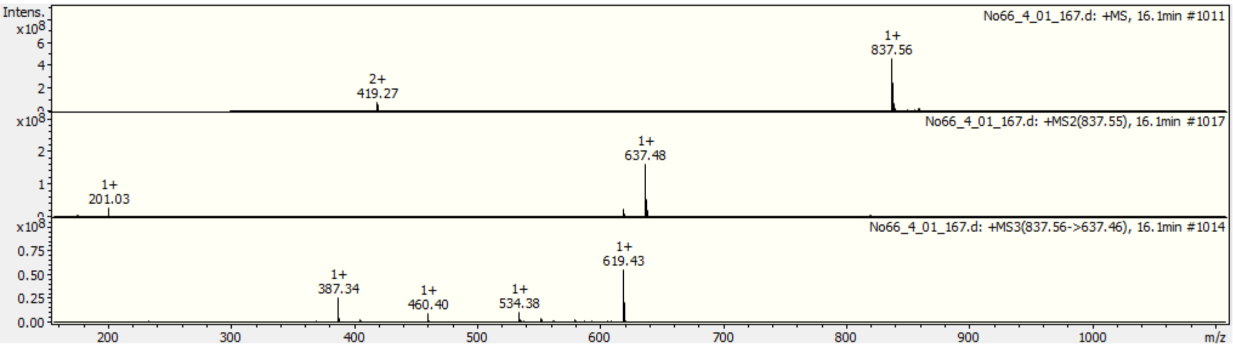

Supplementary Figure S1. Fragmentation pattern of anabaenopeptin B ($[M+H]^+$ 837.5) from strain no. 66.

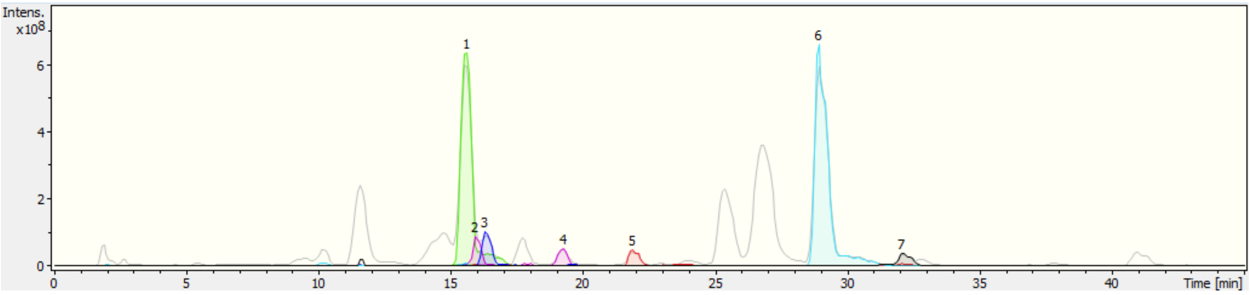

Peak	RT [min]	Area	Area %	m/z	Peptide
1	15.6	2E+10	87	809.5	AP C
2	16.0	2E+09	8	823.5	Unknown AP
3	16.3	2E+09	10	837.5	AP B
4	19.3	1E+09	6	823.5	Unknown AP
5	21.9	1E+09	6	844.5	AP A
6	28.9	2E+10	100	828.5	AP D
7	32.1	1E+09	5	842.5	Unknown AP

Supplementary Figure S2. HPLC-MS chromatogram of strain no. 804. Peaks from anabaenopeptins are colored.

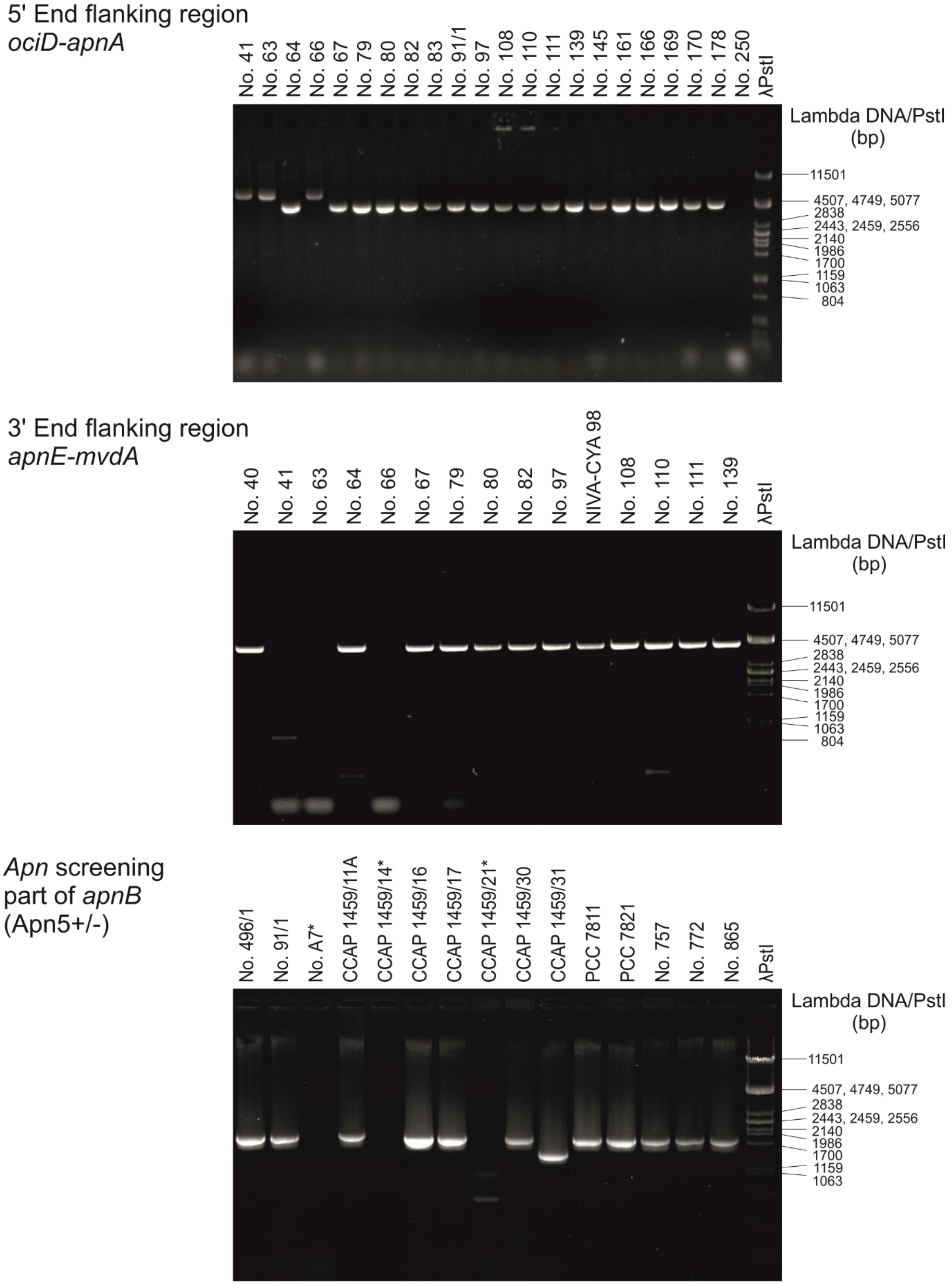

Supplementary Figure S3. Gel pictures showing PCR products of amplified **(A)** *ociD-apnA* flanking region, 4.7 kbp (three strains no. 66, 63 and 41 showed a bigger PCR product due to insertion of IS1634, 2,063 bp), **(B)** *apnE-mvdA* flanking region, 4.4 kbp which was amplified less frequently among strains of lineage 1, **(C)** *apnB* gene fragments with one strain (CCAP 1459/31) carrying a 381 bp deletion. (*, denotes strains which were lost during this study).

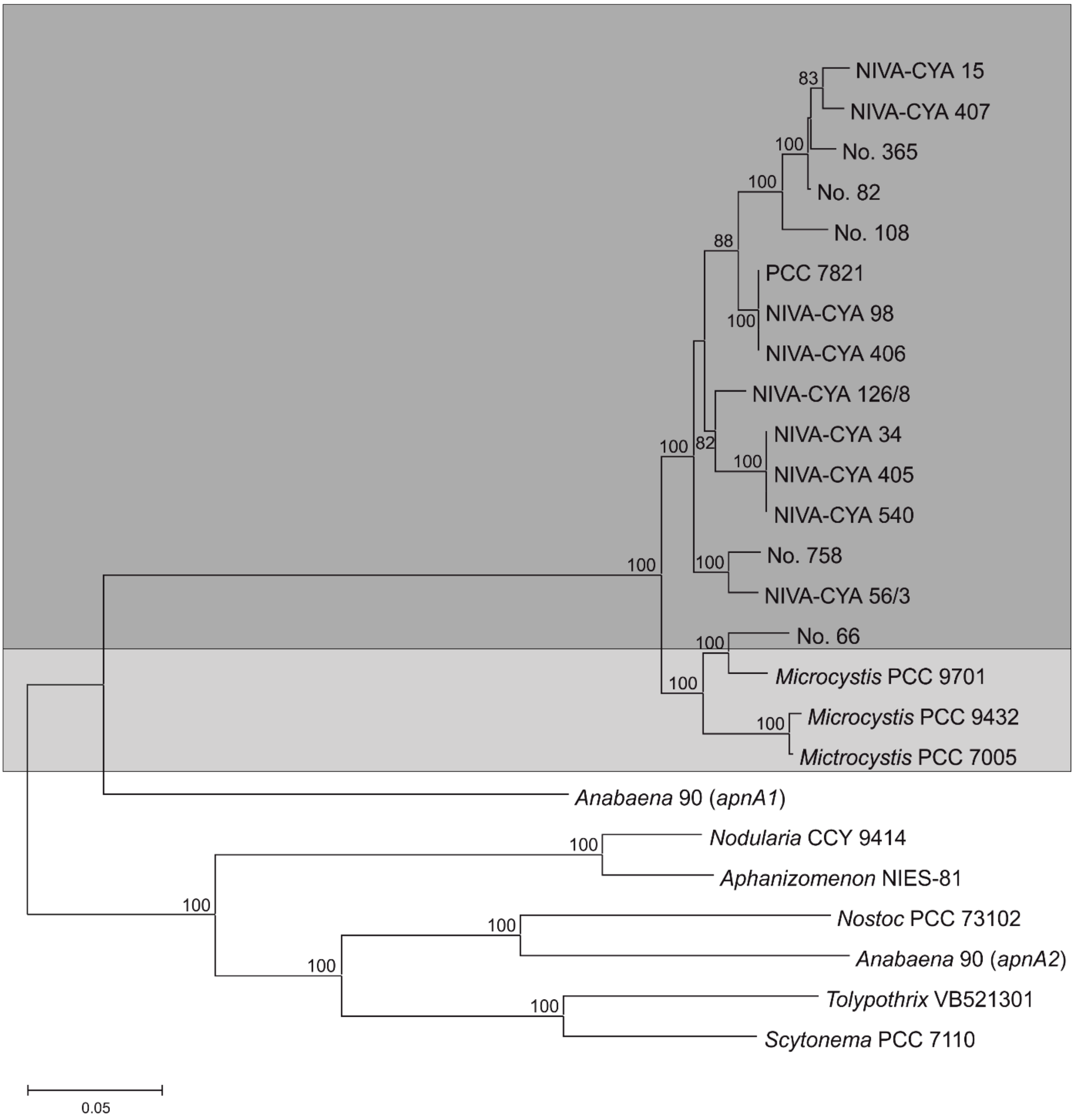

Supplementary Figure S4. Maximum likelihood tree calculated from *apnA* gene sequences (7,114 bp) from *Planktothrix* strains and other cyanobacteria genera.

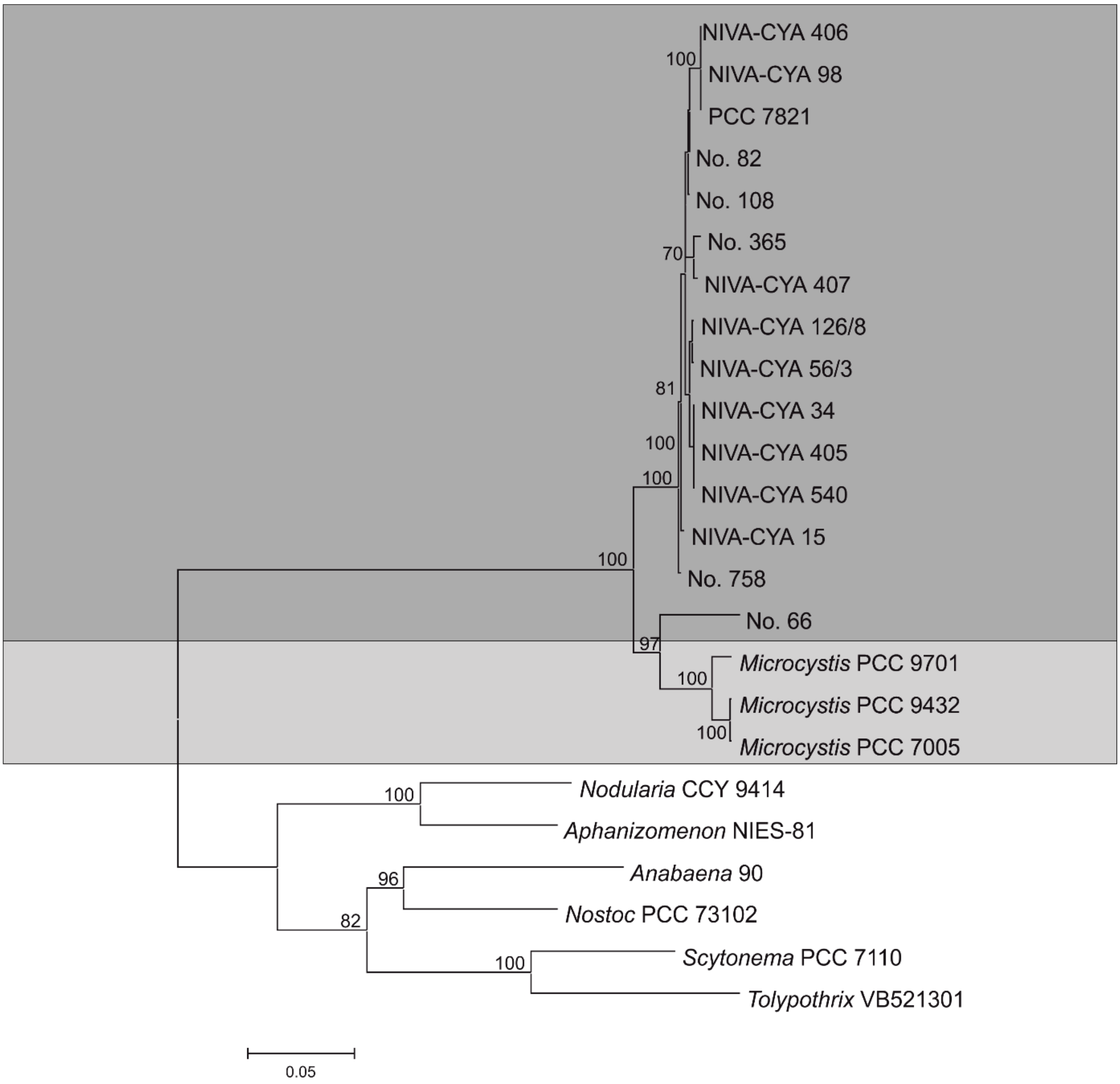

Supplementary Figure S5. Maximum likelihood tree calculated from *apnB* gene sequences (3,285 bp) from *Planktothrix* strains and other cyanobacteria genera.

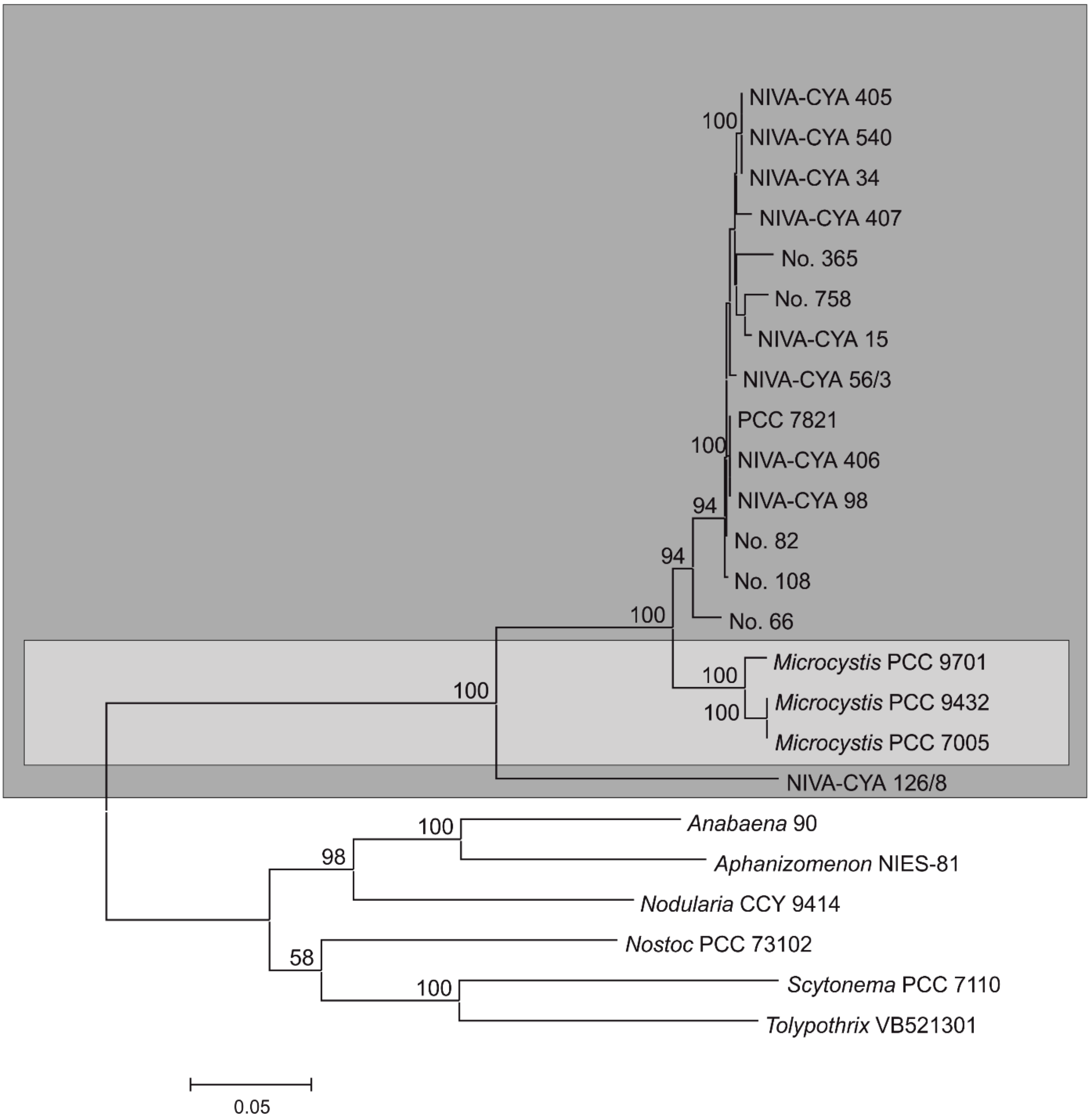

Supplementary Figure S6. Maximum likelihood tree calculated from *apnC* gene sequences (8,202 bp) from *Planktothrix* strains and other cyanobacteria genera.

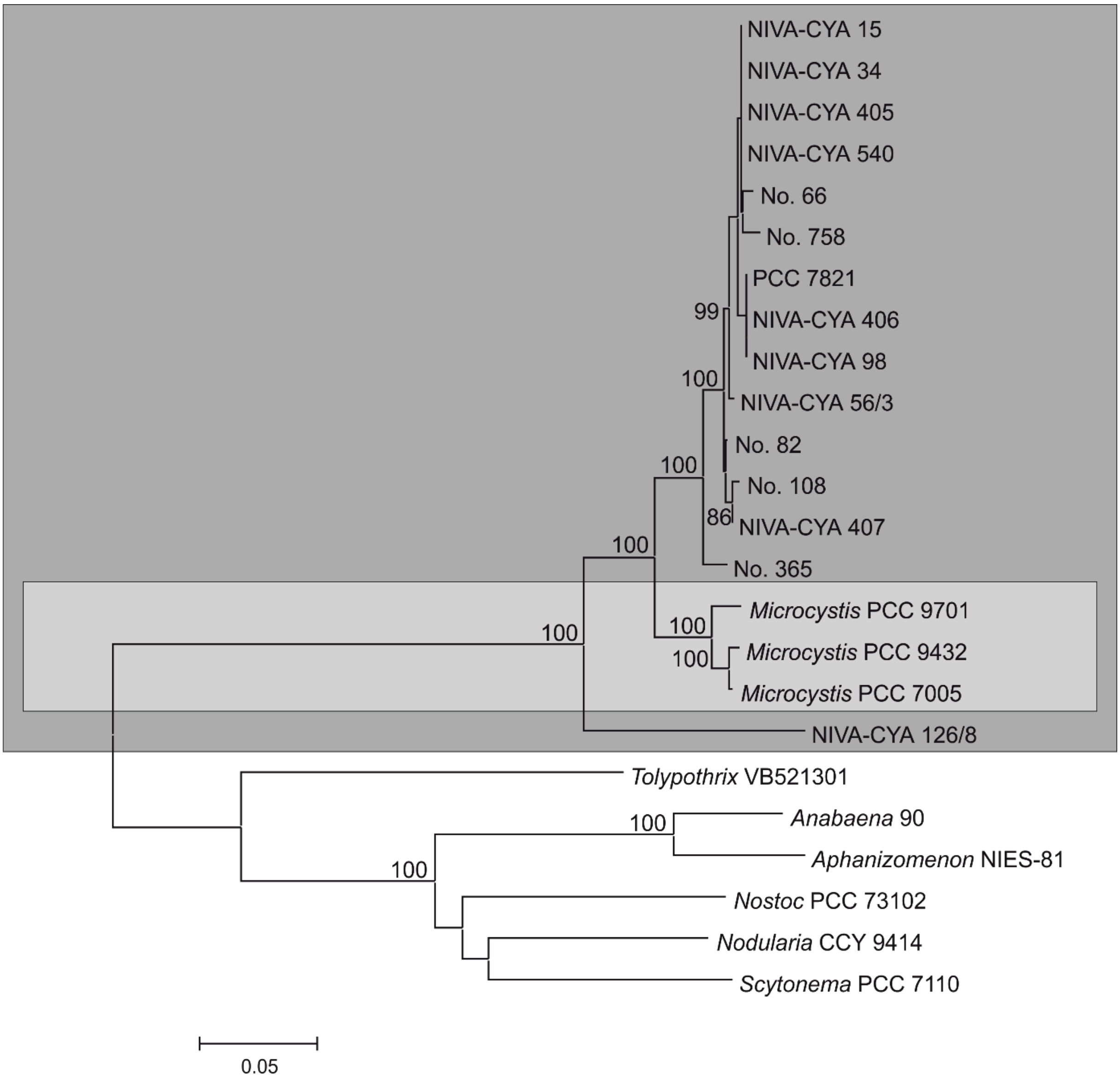

Supplementary Figure S7. Maximum likelihood tree calculated from *apnD* gene sequences (5,050 bp) from *Planktothrix* strains and other cyanobacteria genera.

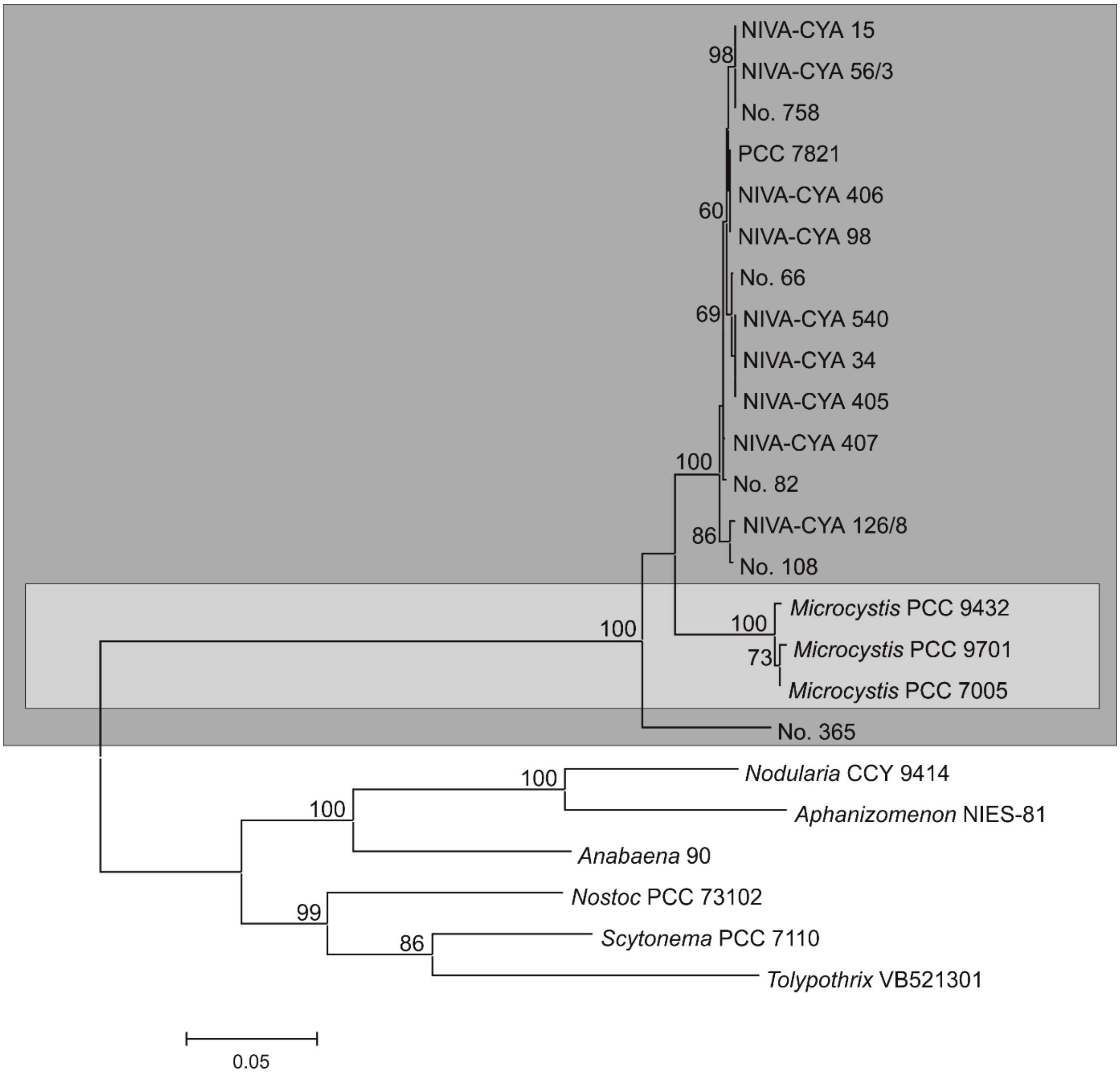

Supplementary Figure S8. Maximum likelihood tree calculated from *apnE* gene sequences (2,339 bp) from *Planktothrix* strains and other cyanobacteria genera.

```
                                                            277/278
No.66               FDVAAEEIYVTLCSGATLILRTEEMISSIPLFVQKSQDWQITVWDLPTAYWHLFVNELVK 258
No.39               FDVAAEEIYVTLCSGATLILRTEEMISSIPLFVQKSQDWQITIWDLPTAYWHLLVNELVN 258
No.64               FDVAAEEIYVTLCSGATLILRTEEMISSIPSFVQKSQDWQITVWSLPTAYWHLFVNELVK 258
No.770              FDVAAEEIYVTLCSGATLILRTEEMISSIPSFVQKSQDWQITVWDLPTAYWHLLVNELVN 258
No.108              FDVAAEEIYVTLCSGATLILRTEEMISSIPSFVQKSQDWRITVIDLPTAYWHLLVNELAN 258
No.804              FDMAAEEIYVTLCSGATLVLRTEEMISSIPLFVQKSQDWRITVLDLPTAYWHLLVNELIN 258
No.371              FDVAAEEIYVTLCSGATLVLRTEEMISSIPLFVQKSQDWQITVWSLPTAYWHLLVNELAN 258
No.396              FDVAAEEIYVTLCSGATLVLRTEEMISSIPLFVQKSQDWQITVWSLPTAYWHLLVNELAN 258
No.67               FDVAAEEIYVTLCSGATLILRTEEMISSIPSFVQKSQDWRITVIDLPTAYWHLLVNELAN 258
No.365              FDVAAEEIYVTLCSGATLVLRTEEMISSIPLFVQKSQDWQITVWSLPTAYWHLLVNELVN 258
No.277              FDVAAEEIYVTLCSGATLILRTEEMISSIPSFVQKSQDWQITVWSLPTAYWHLLVNELVK 258
NIVA-CYA126/8       FDVAAEEIYVTLCSGATLILRTEEMISSIPSFVQKSQDWQITVWSLPTAYWHLLVNELVK 258
No.260              FDVAAEEIYVTLCSGATLILRTEEMISSIPSFVQKSQDWQITVWSLPTAYWHLLVNELVK 258
No.32               FDVAAEEIYVTLCSGATLILRTEEMISSIPSFVQKSQDWQITVWSLPTAYWHLLVNELVK 258
No.3                FDVAAEEIYVTLCSGATLILRTEEMISSIPSFVQKSQDWQITVWSLPTAYWHLLVNELVK 258
No.82               FDVAAEEIYVTLCSGATLILRTEEMISSIPSFVQKSQDWQITVWSLPTAYWHLFVNELVK 258
PCC7821             FDVAAEEIYVTLCSGATLILRTEEMISSIPSFVQKSQDWQITVWSLPTAYWHLLVNELVK 258
No.31/1             FDVAAEEIYVTLCSGATLILRTEEMISSIPSFVQKSQDWQITVWSLPTAYWHLLVNELVK 258
CCAP1459/36         FDVAAEEIYVTLCSGATLILRTEEMISSIPSFVQKSQDWQITVWSLPTAYWHLLVNELVK 258
CCAP1459/17         FDVAVHEVCTTLCSGAALVLRTEEMISSIPSFVQKSQDWQITVWSLPTAYWHLLVNELVK 258
No.806              FDVAAHEIYTTLCSGATLVLRTEEMISSIPSFVQKSQDWQITVWDLPTAYWHLLINELVK 258
GrsA                FDASVWEMFMALLTGASLYIILKDTINDFVKFEQYINQKEITVITLPPTYVVHLDPERI- 292
                    ** :. *:  :* :**:* :  :: *..:  * *  :: .**:  ** :*   :  *

                                                          322      311
No.66               SKIALPNSLRLVIIGGERVQPELVRMWFKNVGDFPELINAYGPTEGTIEVSLCRLSQLTQ 318
No.39               SKIALPDSLRLVIIGGERVQAEIVRMWFKNVGNFPELINAYGPTEGTVEVSVCRLSQLTE 318
No.64               SKIALPDSLRLVIIGGERVQPELVRMWFKNVGNFPELINAYGPTEGTIEVSLCHLSQLTE 318
No.770              SKIVLPDSLRLVIIGGERVQPELVRMWFKNVGNFPELINGYGPTEGTVEVSVCRLSQLTE 318
No.108              SKLVLPGSLRLIIIGGERVQPELVRMWFKNIGNFPELINGYGPTEGTIEVSLCRLSQLTE 318
No.804              SKIALPDSLRLIITGGERIQPELVRVWFKNIGNFPELINGYGPTEGTIEVSLCRLSQLTE 318
No.371              SKLVLPGSLRLIIIGGERVQPELVRMWFKNIGNFPELINGYGPTEGTIAVSLCRLSQLTE 318
No.396              SKLVLPGSLRLIIIGGERVQPELVRMWFKNIGNFPEIINGYGPTEGTIAVSLCRLSQLTE 318
No.67               SKLVLPGSLRLVIVGGERIQPELVRMWFKNVGNFPELINIYGPTEGTIAVSLCRLSQLTE 318
No.365              SKIALPDSLRLVIIGGERVQPELVRLWLKNVGDFPELINAYGPTEGTIAVSLCRLSQLTE 318
No.277              SKIALPDSLRLVIIGGERVQPELVRMWFKNVGNFPELINAYGPTEGTIAVSLCRLSQLAE 318
NIVA-CYA126/8       SKIALPDSLRLVIIGGERVQPELVRMWFKNVGNFPELINAYGPTEGTIAVSLCRLSQLAE 318
No.260              SKIAFPDSLRLVIIGGERVQPELVRMWFKNVGNFPELINAYGPTEGTIAVSLCRLSQLAE 318
No.32               SKIALPDSLRLVIIGGERVQPELVRMWFKNVGNFPELINAYGPTEGTIAVSLCRLSQLAE 318
No.3                SKIALPDSLRLVIIGGERVQPELVRMWFKNVGNFPELINAYGPTEGTIAVSLCRLSQLAE 318
No.82               SKIALPDSLRLVIIGGERVQPELVRMWFKNVGNFPELINVYGPTEGTIAVSLCRLSQLTE 318
PCC7821             SKIALPDSLRLVIIGGERVQPELVRMWFKNVGNFPELINVYGPTEGTIAVSLCRLSQLTE 318
No.31/1             SKIALPDSLRLVIIGGERVQPELVRMWFKNVEIFLELINVYGPTEGTIAVSLCRLSQLTE 318
CCAP1459/36         SKMALPDSLRLVIIGGERVQPELVRMWFKNVGNFPELINVYGPTEGTIAVSLCRLSQLTE 318
CCAP1459/17         SKIALPDSLRLVIIGGERVQPELVRMWFKNVGNFPELINAYGPTEGTIEVSICHLSQLTE 318
No.806              SKVALPDSLRLIIIGGERVQPELVRMWFKNVGNFPELINAYGPTEGTIEVSICHLSQLTE 318
GrsA                ------LSIQTLITAGSATSPSLVNKWKEKV----TYINAYGPTETTICATTWVATKETI 342
                           *:: :* .*.  . .:*. * :::      ** ***** *:    *  : ::

No.66               SQRNRTEIPIGKSLGENISVYVLDETLKTVPPETPGEIYIGGAAVARGY----------- 367
No.39               SQRNRTEIPIGKSLGENISVYVLDETLKIVPPETPGEIYIGGAAVARGY----------- 367
No.64               SQRNRTEIPIGKSLGENISVYVLDETLKIVPTETPGEIYIGGAAVARGY----------- 367
No.770              SQRNRTEIPIGKSLGENISVYVLDETLKIVPPETSGEIYIGGAAVARGY----------- 367
No.108              SQRSRAEIPIGKSLGENISIYVLDETLKIVPPETPGEIYIGGAAVARGY----------- 367
No.804              SQRNRAEIPIGKSLGENISIYVLDETLKIVPPETPGEIYIGGAAVARGY----------- 367
No.371              SQRNRAEIPIGKSLGENISIYVLDETLKIVPSETPGEIYIGGAAVARGY----------- 367
No.396              SQRNRAEIPIGKSLGENISIYVLDETLKIVPSETPGEIYIGGAAVARGY----------- 367
No.67               SQRNRTEIPIGKSLGESISVYVLDETLKIVPPETPGEIYIGGTAVARGY----------- 367
No.365              SQRNRTEIPIGKSLGENISVYVLDETLKTVPPETPGEIYIGGTALARGY----------- 367
No.277              SQRNRTEIPIGKSLGENISVYVLDETLKTVPPETPGEIYIGGTALARGY----------- 367
NIVA-CYA126/8       SQRNRTEIPIGKSLGENISVYVLDETLKTVSPETPGEIYIGGTAVARGY----------- 367
No.260              SQRNRTEIPIGKSLGENISVYVLDETLKTVSPETPGEIYIGGTAVARGY----------- 367
No.32               SQRNRTEIPIGKSLGENISVYVLDETLKIVPPETPGEIYIGGAAVARGY----------- 367
No.3                SQRNRTEIPIGKSLGENISVYVLDETLKIVPPETSGEIYIGGAAVARGY----------- 367
No.82               SQRNRTEIPIGKSLGENISVYVLDETLKTVPPETPGEIYIGGTALARGY----------- 367
PCC7821             SQRNRTEIPIGKSLGENISVYVLDETLKTVPPETPGEIYIGGTALARGY----------- 367
No.31/1             SQRNRTEIPIGKSLGENISVYVLDETLKTVPPETPGEIYIGGTALARGY----------- 367
CCAP1459/36         SQRNRTEIPIGKSLGENISVYVLDETLKTVPPETPGEIYIGGTAVARGY----------- 367
CCAP1459/17         SQRNRTEIPIGKSLGENISVYVLDETLKIVPTETPGEIYIGGAAVARGY----------- 367
No.806              SQRNRTEIPIGKSLGENISVYVLDETLKIVPPETPGEIYIGGAAVARGY----------- 367
GrsA                GHS----VPIGAPI-QNTQIYIVDENLQLKSVGEAGELCIGGEGLARGYWKRPELTSQKF 397
                    .:    .:*** : :. .:*::**.*:       **: *** .:****
```

Supplementary Figure S9. Alignment of *Apn*A A_1-domain sequences (A4-A6, 170 aa) of 21 *Planktothrix* spp. genotypes and indication of positively selected sites as estimated by PAML and Bayes empirical Bayes (BEB) inference (Yang et al., 2005). Red = specificity-conferring Code (Stachelhaus et al., 1999), green = residues within 8 Å around the substrate as defined by Rausch et al. (2005), grey = indicated by BEB analysis (> 90%), numbering according to Stachelhaus et al. (1999).

References

Fujii, K., Sivonen, K., Nakano, T., Harada, K-I. (2002). Structural elucidation of cyanobacterial peptides encoded by peptide synthetase gene in *Anabaena* species. *Tetrahedron 58*:6863-6871. doi: 10.1016/S0040-4020(02)00747-0.

Harada, K-I., Fujii, K., Shimada, T., Suzuki, M., Sano, H., Adachi, K., Carmichael, W.W. 1995. Two cyclic peptides, anabaenopeptins, a third group of bioactive compounds from the cyanobacterium *Anabaena flos-aquae* NRC 525-17. *Tetrahedron Lett. 36*:1511-1514. doi: 10.1016/0040-4039(95)00073-L.

Okumura, H.S., Philmus, B., Portmann, C., Hemscheidt, T.K. (2009). Homotyrosine-containing cyanopeptolins 880 and 960 and anabaenopeptins 908 and 915 from *Planktothrix agardhii* CYA 126/8. *J. Nat. Prod. 72*:172-176. doi: 10.1021/np800557m.

Sano, T., Kaya, K. (1995). Oscillamide Y, a chymotrypsin inhibitor from toxic *Oscillatoria agardhii*. *Tetrahedron Lett. 36*:5933-5936. doi: 10.1016/0040-4039(95)01198-Q.

Rausch, C., Weber, T., Kohlbacher, O., Wohlleben, W., and Huson, D. (2005). Specificity prediction of adenylation domains in nonribosomal peptide synthetases (NRPS) using transductive support vector machines (TSVMs). *Nucl. Acids Res. 33*: 5799-5808. doi: 10.1093/nar/gki885

Stachelhaus, T., Mootz, H.D., and Marahiel, M.A. (1999). The specificity-conferring code of adenylation domains in nonribosomal peptide synthetases. *Chem. Biol. 6*: 493-505.doi: 10.1016/S1074-5521(99)80082-9.

Sano, T., Usui, T., Ueda, K., Osada, H., Kaya, K. (2001). Isolation of new protein phosphatase inhibitors from two cyanobacteria species, *Planktothrix* spp. *J. Nat. Prod. 64*:1052-1055. doi: 10.1021/np0005356.

Shin, H.J., Matsuda, H., Murakami, M., Yamaguchi, K. (1997). Anabaenopeptins E and F, two new cyclic peptides from the cyanobacterium *Oscillatoria agardhii* (NIES 204). *J. Nat. Prod. 60*:139-141. doi: 10.1021/np960597p.

Yang, Z., Wong, W.S., and Nielsen, R. (2005). Bayes empirical bayes inference of amino acid sites under positive selection. *Mol. Biol. Evol. 22*: 1107-1118. doi: 10.1093/molbev/msi097.
